# Electrochemical Switch‐On of Nonlinear Optical Response in a Nitro‐Functionalized Arylimido‐Polyoxometalate

**DOI:** 10.1002/anie.4949539

**Published:** 2026-06-30

**Authors:** Claire F. Jones, Bethany R. Hood, Yovan de Coene, Hayleigh Kearns, Karen Faulds, Benoît Champagne, Koen Clays, John Fielden

**Affiliations:** ^1^ School of Chemistry Pharmacy and Pharmacology University of East Anglia Norwich UK; ^2^ School of Chemistry University of East Anglia Norwich UK; ^3^ Department of Chemistry Lancaster University Lancaster UK; ^4^ Department of Chemistry University of Leuven Leuven Belgium; ^5^ Department of Pure and Applied Chemistry University of Strathclyde Glasgow UK; ^6^ Laboratory of Theoretical Chemistry Unit of Theoretical and Structural Physical Chemistry NISM (Namur Institute of Structured Matter) University of Namur Namur Belgium

**Keywords:** Donor‐acceptor systems, Molecular switches, Nonlinear Optics, Polyoxometalates, Spectroelectrochemistry

## Abstract

Electrochemical switch‐on of second‐order non‐linear optical (NLO) responses (*β*) has been demonstrated in a polyoxometalate (POM) based chromophore (POMophore) for the first time. Reduction of the POM in a POM‐imidoaryl‐NO_2_ derivative invokes an up to 15‐fold off/on switchable increase in intensity of scattered frequency‐doubled light, and the highest “on” state *β* yet reported for a redox‐cyclable POMophore. Computational and spectroscopic studies show that in the oxidized state, directionally opposed charge transfer (CT) transitions to POM and ─NO_2_ result in a low net *β*. Upon reduction, the POM becomes a much weaker CT acceptor, resulting in more dipolar imido‐aryl to nitro CT, and thus enhanced NLO response.

## Graphical abstract

Reducing a nitro‐derivatized arylimido‐polyoxometalate (POM) reversibly switches on its second‐order NLO response, contrasting with prior donor‐functionalized systems that switch off upon reduction. Attenuation of charge‐transfer (CT) to the POM upon reduction gives a more strongly dipolar imido/π‐bridge to ─NO_2_ CT, signal enhancement of up to 15× (*β ca*. 4×), and the highest multi‐cycle on‐state activity yet seen for such compounds.

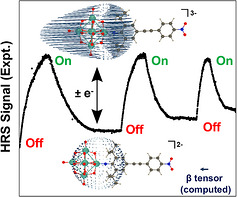

## 1 Introduction

Molecules and molecule‐based materials whose properties switch in response to an external stimulus are vital to many current and future applications: sensors and imaging agents [1, 2, 3, 4], high‐density resistive memory [5, 6], and optical/electro‐optical switches [7] that could underpin new high‐efficiency approaches to computing. For optical computing, the instantaneous nature of molecular nonlinear optical (NLO) responses [8, 9, 10, 11] makes molecules with switchable second‐order NLO attractive for fast data processing and storage. Such molecular NLO materials comprise organic or metallo‐organic charge transfer (CT) chromophores, generally with dipolar donor(D)‐π‐acceptor(A) systems where NLO activity arises from electronic displacements in response to light [8, 9, 10, 11, 12, 13, 14, 15, 16, 17, 18]. Molecular assessment of performance through measurement of first hyperpolarizability *β* (and extrapolation to zero‐frequency giving static *β*_0_) has revealed molecular design rules to optimize response [19, 20, 21, 22, 23, 24, 25, 26], and on/off switching can be achieved by disrupting the D‐π‐A system through redox chemistry, pH changes, or photoisomerisation [27, 28, 29, 30, 31, 32, 33, 34, 35, 36, 37, 38, 39, 40]. Of these, electrochemical redox switching is particularly attractive, due to its speed and potential for device integration. It has been reported a handful of times, mostly in metallo‐organic complexes with redox‐active precious metal donors (e.g., Ru^II^) [37, 38, 39, 40]. However, we are still some distance from achieving switchable molecular NLO materials with the required speed, contrast and stability.

Having multiple, accessible redox states and generally fast, stable redox chemistry [41], POMs are attractive candidates for molecular redox‐switching that have shown promise for use in electronic memory technologies [42, 43]. Yet, investigation of POMs as switched optical and nonlinear optical (NLO) materials has been more limited [44, 45, 46]. We have been investigating POM acceptors—strongly conjugated to aryl groups through an imido bond—as an approach to switchable NLO chromophores based on higher abundance elements, and without the undesirable low‐energy absorptions introduced by Ru^II^ [45, 46, 47, 48, 49, 50]. With organic donors, these can achieve high activities (*β*_0,zzz_ up to 320×10^−30^ esu) [45], and cyclable electrochemical switch‐off has been achieved by reduction of the POM acceptor [46]. Encouraged by prior computational work showing reduced POMs acting as electron donors toward aryl groups [51], we decided to explore the properties of POMs derivatised with nitro‐aromatic acceptors in both oxidised ([Mo_6_O_18_NAr]^2−^) and reduced ([Mo_6_O_18_NAr]^3−^) states.

Herein, we show for the first time a POM chromophore whose NLO activity switches on upon electrochemical reduction. One‐electron reduction of acceptor(A)‐π‐A nitro derivative [Mo_6_O_18_N(^i^Pr)_2_PhCCPhNO_2_]^2−^ ([**1**]^2−^) produces a 15‐fold increase in the measured in situ hyper‐Rayleigh Scattering (HRS) intensity, indicating a *β‐*response comparable to previous D‐π‐A POMophores with strong organic donors, and higher than any other POMophore able to redox‐switch over multiple cycles [16]. As the gained electron is hosted in a POM‐based SOMO isolated from the π‐system, the red shift in the dominant charge transfer (CT) transition is only 11 nm, and unexpectedly the switch‐on can be considered to result from turn‐off of the POM acceptor enabling CT toward ‐NO_2_ from an electron‐rich imido‐diphenylacetylene, rather than direct participation of the POM as an electron donor.

## 2 Results and Discussion

**FIGURE 1:**
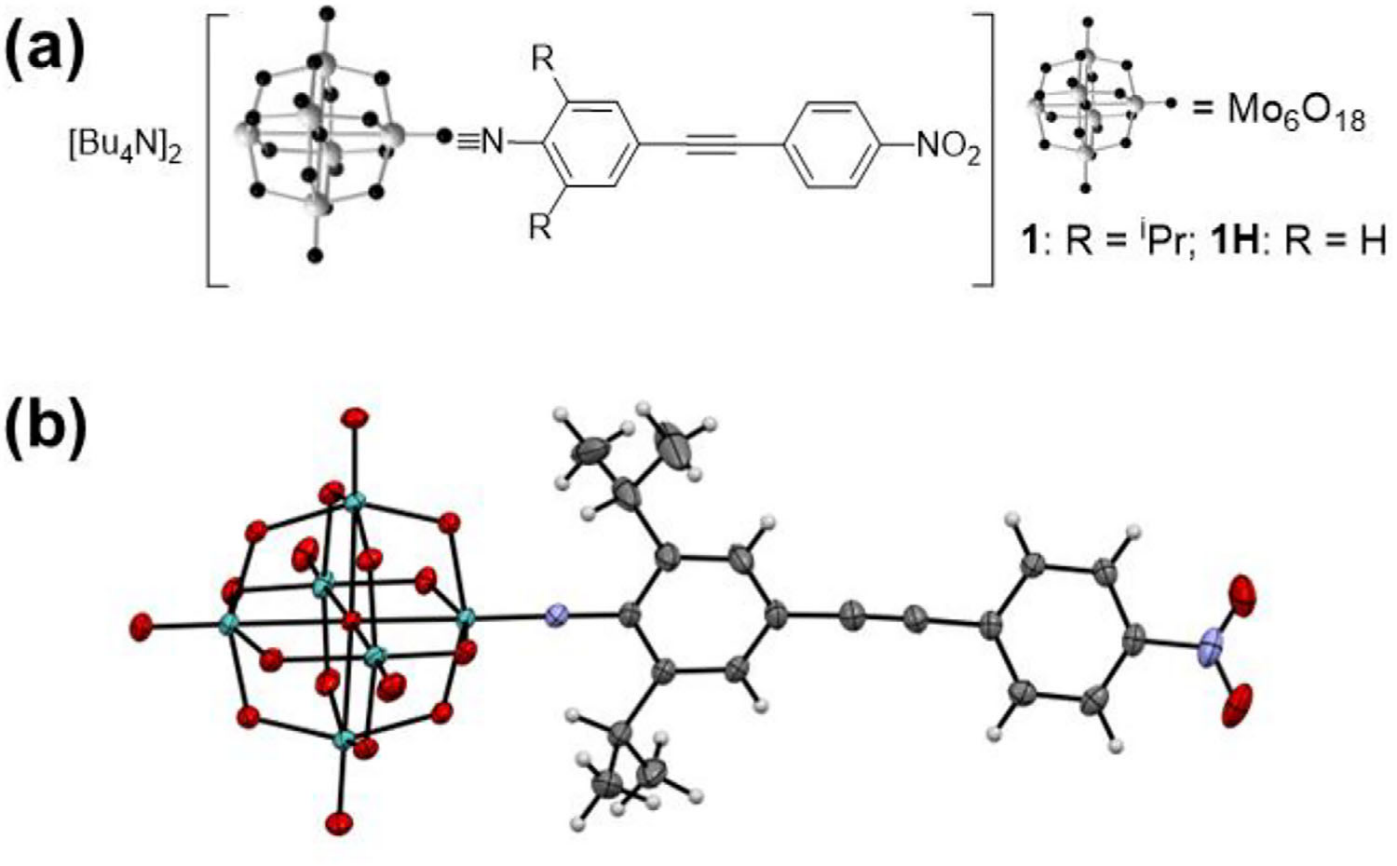
(a) Structures of [**1**]^2−^ and previously published isopropyl‐free analog [**1H**]^2−^ [48]. (b) ORTEP representation of the crystal structure of anion [**1**]^2−^. C is grey; N, blue; O, red; Mo, green. H atoms are white spheres of arbitrary radii. Thermal ellipsoids drawn at the 30% probability level.

[NBu_4_]_2_[**1**] (Figure 1) was obtained from [NBu_4_]_2_[Mo_6_O_19_] following similar methods to those previously used for isopropyl‐free analog [NBu_4_][**1H**] (Scheme S1, SI) [48], and thoroughly characterized (see SI for details), including a high‐quality X‐ray crystal structure (Figures 1b and S1; Tables S1 and S2). Mo─O bond lengths were comparable to those seen in [**1H**]^2−^ and other [Mo_6_O_18_NAr]^2−^ clusters [45, 46, 47, 48, 49, 50], while Mo─N (ca. 1.74 Å) and N─C (ca. 1.38 Å) distances were within experimental errors of those reported for ^i^Pr‐free systems, though considerably more linear (176.0(2)° and 173.9(4)°) due to the bulky isopropyl groups. Despite low energy barriers to rotation in unhindered phenylacetylenes [52, 53, 54], previous work with arylimido‐POMs [45, 46, 47, 48, 49, 50] has shown increasing coplanarity of the phenyl rings with weaker donor groups, and with the presence of 2,6‐isopropyl groups on the imido‐ring. Anion [**1**]^2−^ fits this pattern, with the lowest torsion angle across the alkyne (10.44°) of any derivative we have studied so far, consistent with lower charge asymmetry between the two phenyl groups strengthening conjugation. No quinoidal character is seen in [**1**]^2−^, as expected for essentially asymmetric A‐π‐A rather than D‐π‐A systems.

**FIGURE 2:**
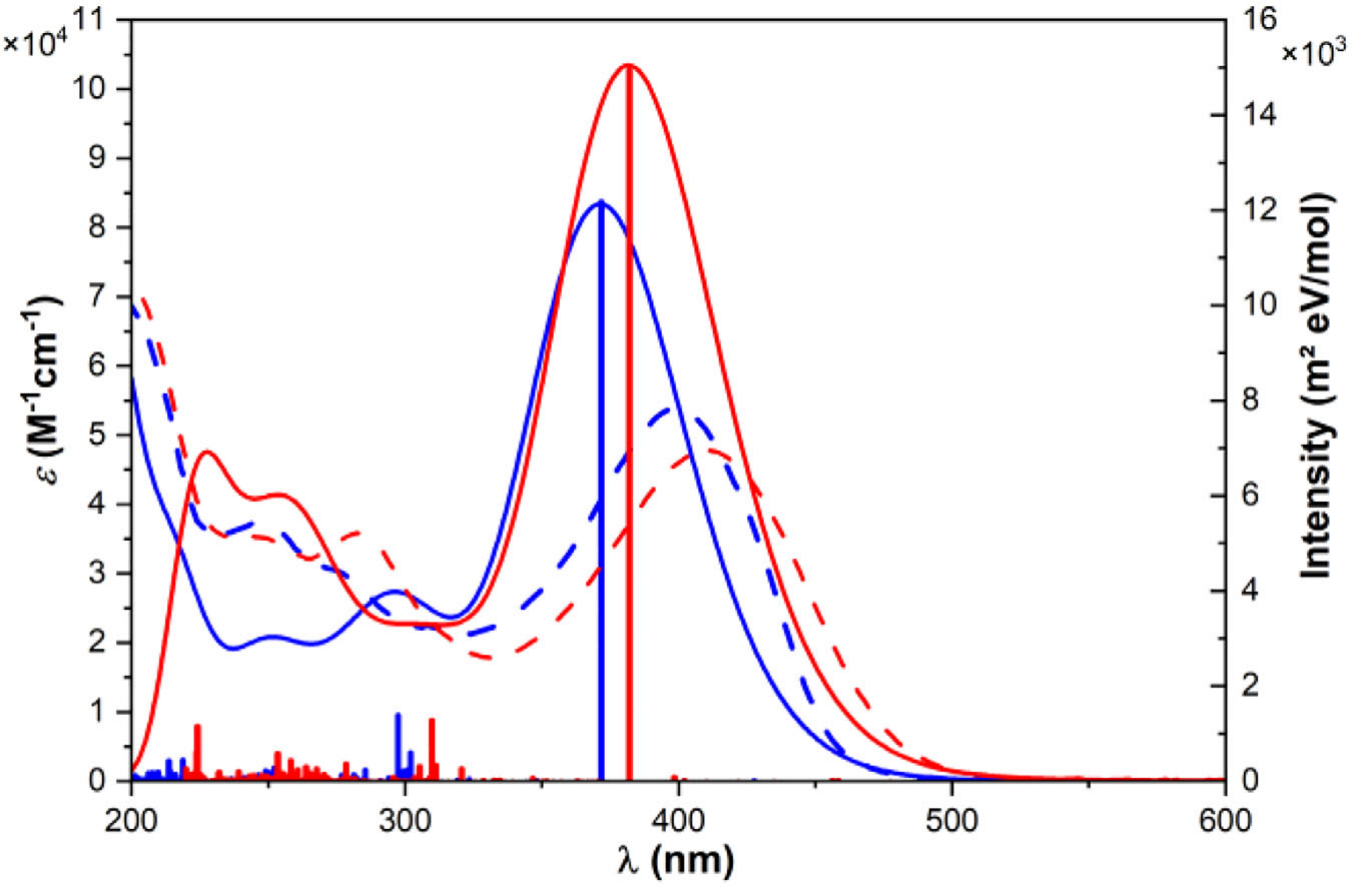
Experimental (dashed lines) and computed (solid lines) UV‐vis spectra of [**1**]^2−^ (blue) and [**1**]^3−^ (red). Vertical bars represent computed vertical transitions. The spectrum of [**1**]^3−^ was obtained by spectroelectrochemistry (electrolyte 0.3 M NBu_4_BF_4_ in MeCN), original spectrum was 98% recovered upon reoxidation. Calculations performed at the IEFPCM (MeCN) TD‐DFT/ωB97X‐D/6‐311G(d)/LanL2TZ level of theory.

**FIGURE 3:**
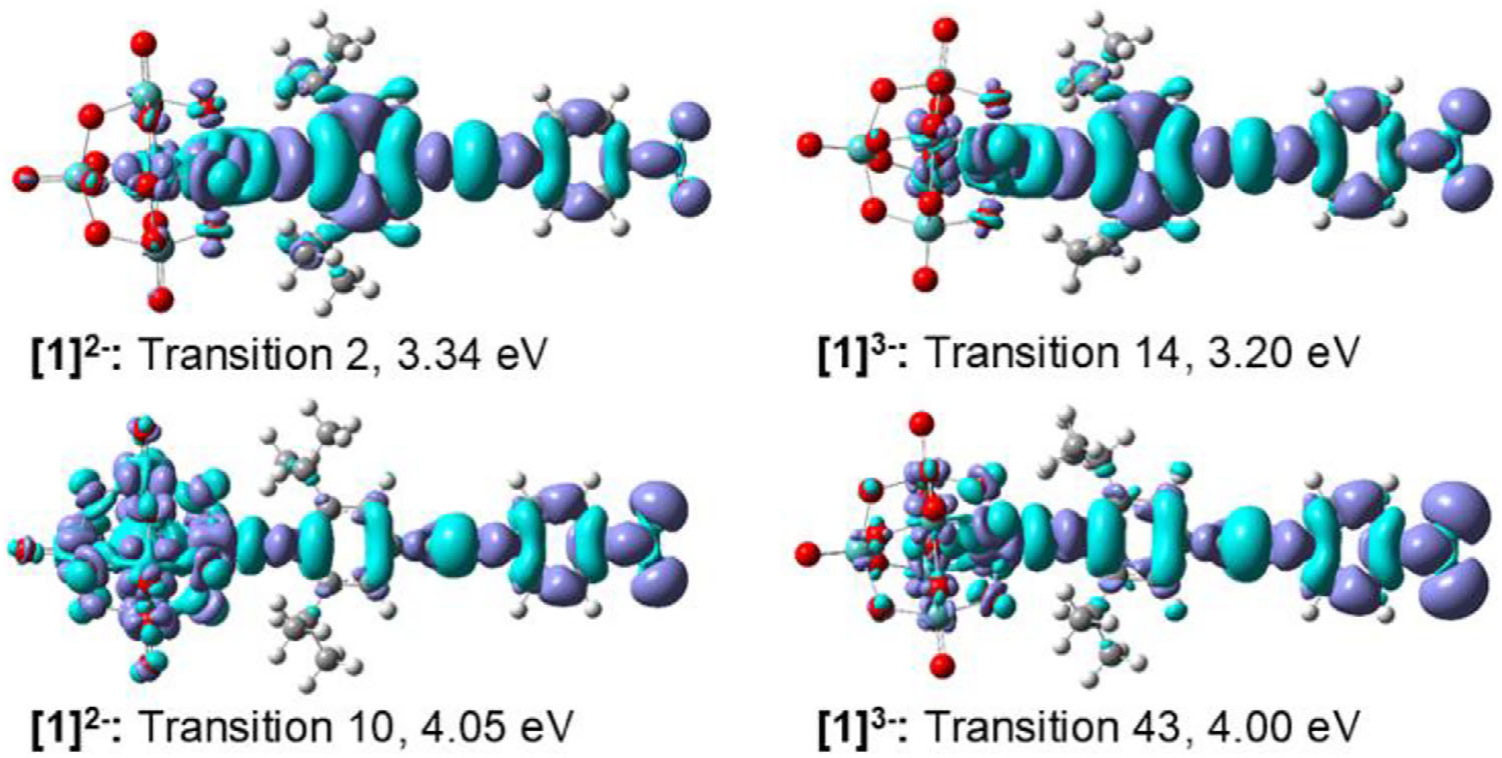
Changes of electron density, ∆𝜌 = 𝜌_𝑒_ − 𝜌_𝑔_ between the ground (GS) and dominant charge‐transfer excited (ES) states of [**1**]^2−^ and [**1**]^3−^. Isovalues 0.0004 a.u., color map: cyan represents negative ∆𝜌, blue positive ∆𝜌.

The UV–vis spectrum of [**1**]^2−^ (Figure 2, Table 1) shows several absorptions in the 200–300 nm region, and a lower energy peak at 399 nm. TD‐DFT calculations indicate π→NO_2_ transitions and ligand‐to‐POM CT (LPCT) both contribute to the lower energy peak; the red shift of 10 nm (0.07 eV) versus [**1H**]^2‒^ (Figure S5 and Table S4) is thus consistent with the inductive ^i^Pr donors raising the energy of the imido‐aryl donor ring. Competition between the two acceptors is roughly even (Figure 3), but a small positive ground‐to‐excited state dipole moment change *Δµ*_GE_ (+2.31 D, Table S6) indicates a net shift of electron density toward the POM. Raman measurements at 785 and 532 nm show enhancement of POM and ─NO_2_ bands at the lower wavelength, consistent with the computational picture. TD‐DFT analysis of the higher energy bands (λ < 340 nm), mostly characterized by O→Mo LMCT and π→π* processes, indicates the presence of a weaker transition with LPCT and π→NO_2_ character at 4.05 eV (306 nm), in this case with a larger, negative *Δµ*_GE_ indicating CT toward ─NO_2_.

**TABLE 1:** UV–vis absorption and electrochemical data for [**1**]^2‐/3−^ in MeCN.

	*λ*_max_/nm (*ε*, 10^3^ M^−1^ cm^−1^)		*E*_max_/eV		*E*/V vs. Fc/Fc^+^ (Δ*E*_p_/mV)^d^
Anion	Expt	Calc^c^	Expt	Calc	*E*_1/2_ [**1**]^2‐/3−^
[**1**]^2−^	246 (37.3)^a^ 276 (30.4)^a^ 399 (53.7)^a^	252 (20.8) 296 (27.3) 371 (83.4)	5.04 4.49 3.11	4.92 4.19 3.34	−1.00 (73)
[**1**]^3−^	244 (35.4)^b^ 284 (35.8)^b^ 410 (47.8)^b^	228 (47.5) 255 (41.3) 381 (103)	5.08 4.37 3.02	5.44 4.86 3.25	

^a^ Obtained at ca. 10^−5^ M in MeCN.

^b^ Obtained from ca. 10^−2 ^M solution in 0.3 M [NBu_4_][BF_4_] in a thin layer spectroelectrochemistry cell, with initial [**1**]^2−^ absorbances as a reference.

^c^ IEFPCM (MeCN) TD‐DFT/ωB97X‐D/6‐311G(d)/LanL2TZ.

^d^ ca. 10^−3^ M in analyte, 0.1 M [NBu_4_][BF_4_] in MeCN at a glassy carbon working electrode, scan rate 100 mV s^−1^.

Cyclic voltammetry (Tables 1 and S3; Figure S3) finds two processes assigned to the POM by comparison with known [ArNMo_6_O_18_]^2−^ species: [45, 46, 47, 48, 49, 50] a reversible [**1**]^2‐/3−^ reduction at −1.00 V versus Fc/Fc^+^, and an irreversible process at −1.9 V involving destructive reduction. In between is a quasi‐reversible one‐electron [**1**]^3‐/4−^ process at ‐1.41 V—this is not observed in other [ArNMo_6_O_18_]^2−^ compounds, and is assigned to −NO_2_ reduction due to its similarity to 4‐nitro‐diphenylacetylene [55], and computed spin density and redox potentials of both triplet and singlet diradical [**1**]^4−^ anions (Figure S7 and Table S3). Comparison with [**1H**]**^2−^** (Figure S5 and Table S4) again shows the electron‐donating effect of the ^i^Pr groups, through a 50 mV negative shift in the POM redox potential.

The reversibility of the [**1**]^2‐/3−^ couple encouraged study of [**1**]^3−^. Around 76% of [**1**]^2‐/3−^ was found to persist in solution after 23 min reductive bulk electrolysis (Figure S4)—a huge improvement on ^i^Pr‐free systems that are ca. 80% converted to [Mo_6_O_19_]^2‐/3−^ [46], and adequate for further study of reduced state properties. Low temperature (10 K) EPR measurements (Figure S10) carried out on [**1**]^3−^ find a signal at ca. *g* = 1.92 showing hyperfine coupling to a single Mo centre, but no significant N hyperfine couplings—as for donor‐functionalized arylimido‐POMs [17], the spectrum is little different to that of [Mo_6_O_19_]^3−^, indicating little interaction of the unpaired electron with the ligand. Correspondingly, DFT calculations locate all of the spin density on the POM, and find a POM‐based SOMO (spin‐α HOMO) ca. 0.6 eV above the closely spaced, ligand‐based spin‐α HOMO‐1/spin‐β HOMO (Figure S7). This shows the effect of the ─NO_2_ group in lowering the energy of the π‐orbitals: in donor functionalised systems the POM‐based SOMO is lower in energy than the ligand‐based HOMOs.

UV–vis spectroelectrochemistry (Table 1 and Figures 2 and S11) reveals an 11 nm (0.09 eV) red shift, and slight loss of intensity for the low‐energy transition of [**1**]^3−^ versus [**1**]^2−^, with reversibility demonstrated by 98% recovery of the original spectrum upon reoxidation. The red shift contrasts with blue shifts reported upon reduction for donor functionalized POMophores [45, 46]. TD‐DFT calculations reproduce the red shift (10 nm, 0.09 eV), but find an increased intensity for the transition. Importantly, a negative rather than positive *Δµ_GE_* (Table S6) shows that the net CT direction changes in favour of ─NO_2_ as the POM electron acceptor is weakened by reduction, although the minimal differences in electron density difference plots (Figure 3) and modest amplitudes of *Δµ_GE_* (ca. ± 2 D) indicate that CT is not strongly dipolar in either case. Experimentally, upon reduction, a red shift is also observed in a higher‐energy transition (276 to 284 nm), and TD‐DFT finds the 4.05 eV LPCT/π→NO_2_ transition of [**1**]^2−^ shifts to 4.0 eV in [**1**]^3−^ and loses LPCT character (Figure 3) with *Δµ*_GE_ = −7.06 D indicating CT away from the POM. The small separation observed electrochemically (400 mV, i.e., 0.4 eV) between the POM and ─NO_2_ acceptors prompted investigation of long‐range, low‐energy POM*→*NO_2_ CT transitions by NIR measurements (to 3300 nm). No such transitions were found, nor does TD‐DFT predict them, consistent with the lack of interaction between POM‐based SOMO and ligand shown by EPR. NIR measurements (Figure S11b) did reveal a broad peak centred at around 950 nm, indicating a reduced {Mo_6_} moiety: similar broad, low‐energy IVCT features have been reported in the spectra of [Mo_6_O_19_]^3−^ [56, 57]. The overall picture that emerges is that reduction results in stronger net CT toward ─NO_2_ from the ligand, but the reduced POM does not become a CT donor.

**FIGURE 4:**
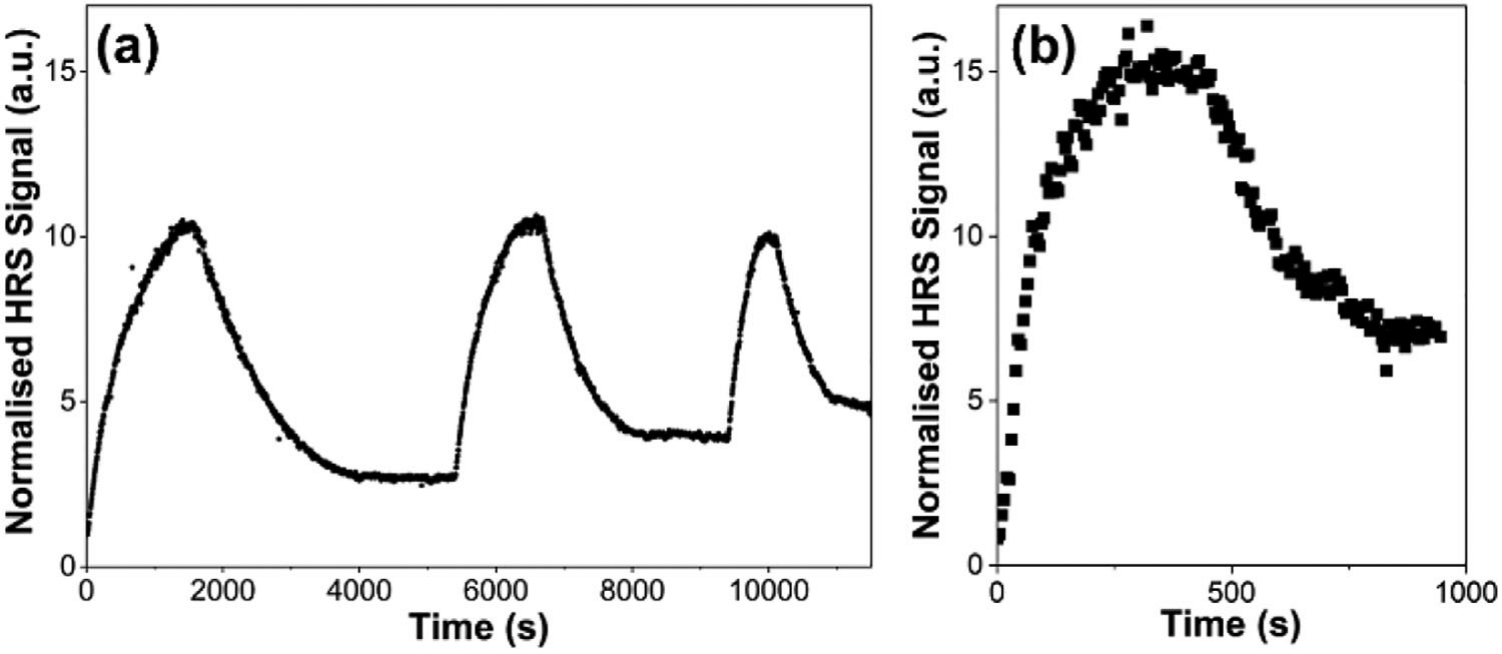
Normalised changes in HRS intensity in: (a) Cycled, electrochemically‐switched HRS experiment using −0.8 V reductive potential. (b) Electrochemically‐switched HRS experiment with −1.1 V reductive potential.

HRS measurements (1064 nm) found a modest first hyperpolarizability for [**1**]^2−^ (*β*_zzz,0_ = 63 × 10^−30^ esu) (Table 2). Upon electrochemical reduction of [**1**]^2−^ to [**1**]^3−^ (‐0.8 V vs. Ag/Ag^+^, avoiding reduction of the ─NO_2_ group), HRS signal intensity augmented (Figure 4a), reaching a >10‐fold increase upon the plateau at 1500 s (0.11 C passed, 60% of [**1**]^2−^ reduced). Three on/off cycles were completed over ca. 3 h, with a slight decrease in the maximum on and larger increase in the maximum off signals. This increase in off signal can be explained by incomplete reoxidation—the 0.068 C passed in the first reoxidation is only sufficient to reduce the signal to around 4× the starting value. The observed post‐cycle 1 off signal of 2.7× thus suggests some decomposition of [**1**]^3−^ to NLO inactive /less active by‐products; the 10× signal is regained due to a reservoir of [**1**]^2−^ (40% unreacted in the first cycle). This is supported by cycled SE showing a *ca*. 20% loss in the low‐energy peak absorbance over 50 min of repeated redox cycling (Figure S10c).

**TABLE 2:** Experimental and computed 1064 nm and static *β*‐responses for [**1**]^2−^ and [**1**]^3−^.

Anion	*β*_zzz,1064_ ^a^ (expt)	*β*_zzz,0_ ^b^ (expt)	*β*_zzz,1064_ ^c^ (calc)	*β*_zzz,0_ ^d^ (calc)
[**1**]^2−^	170	63	6.9	4.4
[**1**]^3−^	670	230	104	40

All values in B convention, units 10–^30^ esu. Calculations performed at the IEFPCM (MeCN) TD‐DFT/ωB97X‐D/6‐311G(d)/LanL2TZ level of theory.

^a^ Dynamic *β*_zzz_ derived from total molecular 1064 nm HRS response assuming a single dominant tensor component.

^b^ Non‐resonant, static *β*_zzz_ estimated from *β*_zzz,1064_ using the two‐state model [59, 60].

^c^ Dynamic HRS *β* response calculated at 1064 nm.

^d^ Static response extracted from calculations at λ = 1500 nm, to avoid resonance effects.

For maximum switch‐on (Figure 4b), a more negative potential was applied (−1.1 V vs. Ag/Ag^+^) to a larger sample, and the HRS signal reached a plateau after 400 s (0.25 C, 97% of [**1**]^2−^ reduced) at >15× its initial value. HRS signal has a linear dependence on the concentration of the active species, and the increased enhancement versus the first cycle in Figure 4a is consistent with the increased percentage of [**1**]^3−^ generated. This implies an estimated *β*_zzz,1064_ of ∼670 × 10^−30^ esu, and *β*_zzz,0_ of ca. 230 × 10^−30^ esu, giving [**1**]^3−^ a similar activity to high‐performing D‐π‐A POMophores, but better visible transparency (λ_max_ = 410 nm), and nearly 3× higher activity than the on‐state of the only other POMophore to demonstrate multi‐cycle redox switching [46].

Full reoxidation again proved challenging, and imperfect stability and switching raise questions about contributions from by‐products. However, observation of >75% [**1**]^2‐/3−^ persisting post bulk electrolysis (Figure S4), and the lower *β*‐values expected for potential by‐products ([Mo_6_O_18_N(^i^Pr)_2_PhCCPhNH_2_]^2−^, [**1R**]^2−^, and 4‐[2‐(4‐nitrophenyl)ethynyl]‐2,6‐diisopropylbenzenamine, **1L**, Figure S13), means that these cannot account for more than a 2× signal switch on versus [**1**]^2−^ (HRS signal ∝ *β* ^2^ and concentration of the active species). Such D‐π‐A structures also have opposite redox‐cycled HRS switching (reduced off/oxidized on) to that observed^15^ – confirmed computationally with *β*_1064,zzz_ = 301 × 10^−30^ esu for [**1R**]^2−^, and only 16 × 10^−30^ esu for [**1R**]^3−^. Thus, while contributions to the signal from NLO active side products cannot be excluded, they cannot explain the magnitude or direction of the observed switching, and as *β* for [**1**]^3−^ has been based on 100% conversion, the generation of these lower *β* side products would result in underestimated rather than overestimated activity for [**1**]^3−^. Electrochemical (Figure S4) and spectroelectrochemical (Figure S11d) evidence, and literature precedent [45], are most consistent with the side products being **1L** and small, NLO and electrochemically inactive molybdates such as [Mo_2_O_7_]^2−^.

TD‐DFT (IEFPCM/ωB97X‐D/6‐311G(d)/LanL2TZ) calculation of *β*‐values broadly reproduces the experimental trend for [**1**]^2−^ and [**1**]^3−^ (Table 2), with unit sphere representations of the *β*‐tensor (Figure S9) illustrating the dramatic change in magnitude between [**1**]^2−^ and [**1**]^3−^, and its opposite direction to that seen in donor‐functionalized imido‐POMs. Activities for both states are underestimated versus experiment, and it should be noted that the method, while generally accurate [58], has also underestimated *β* in donor‐diphenylacetylene imido‐POMs with delocalized or very strong donors [45, 50]. Qualitatively, the increase in *β* from [**1**]^2−^ to [**1**]^3−^ can be understood by considering the computed CT transitions: in [**1**]^2−^ different signs for *Δµ_GE_* indicate that net directions along the molecular axis are opposed, so *β*_zzz_ contributions for each one cancel each other out, but in [**1**]^3−^ the directions are the same, so they reinforce each other. Moreover, using the computed *Δµ_GE_*, oscillator strength (*f_GE_*), and transition energy and the two‐state approximation (TSA) expression, the contribution of selected transitions to *β*_zzz_ can be estimated (Table S4) and summed. Considering two dominant excited states, this better reproduces the experimental result for [**1**]^2−^ (*β*_zzz,0_ = 32×10^−30^ esu) but underestimates the reduced state switch‐on. This is because the ΣTSA model does not include any higher excited states, and lacks terms coupling different excited states [61].

## 3 Conclusion

In conclusion, we have provided the first experimental demonstration that acceptor functionalised arylimido‐POMs—non‐symmetrical acceptor‐π‐acceptor systems—can have significant optical non‐linearities and act as redox‐mediated NLO switches. Upon reduction of the POM, weakened acceptor properties enable the imido‐aryl group to become an effective donor, giving near 4× enhanced second‐order NLO activity *β* (15× enhanced signal) without strong, low‐energy absorptions. This gives exciting perspectives for use of reduced POM‐arylimido units as electron donors in NLO materials, and more generally for using imido‐polyoxometalate redox chemistry to modulate the properties of attached organic π‐systems.

## Author Contributions

**Claire F. Jones**: investigation, writing – review and editing, formal analysis, and writing – original draft. **Bethany R. Hood**: investigation, writing – original draft, and formal analysis. **Yovan de Coene**: investigation, formal analysis, and funding acquisition. **Hayleigh Kearns**: investigation, formal analysis, and writing – review and editing. **Karen Faulds**: funding acquisition and writing – review and editing. **Benoît Champagne**: supervision and writing – review and editing. **Koen Clays**: funding acquisition, writing – review and editing, and supervision. **John Fielden**: funding acquisition, writing – review and editing, writing – original draft, formal analysis, data curation, supervision, and project administration.

## Conflicts of Interest

The authors declare no competing financial interests.

## Supporting information




**Supporting File 1**: anie73359‐sup‐0001‐SuppMat.pdf.

**Supporting File 2**: anie73359‐sup‐0002‐cif.zip.

## Data Availability

Data supporting this paper are presented in the supplementary information and will be deposited at DOI: 10.17635/Lancaster/researchdata/278. The CIF file for the structure of [NBu_4_]_2_[**1**] is available from the CCDC (deposition number 2523544) via https://www.ccdc.cam.ac.uk/structures/.

## References

[1] J. H. Zhu , M. Gu , Y. Chen , et al., “Chemiluminescent Transition Metal Complexes: Mechanisms and Applications,” Coordination Chemistry Reviews 530 (2025): 216495, 10.1016/j.ccr.2025.216495.

[2] Y. Ding , W.‐H. Zhu , and Y. Xie , “Development of Ion Chemosensors Based on Porphyrin Analogues,” Chemical Reviews 117 (2016): 2203–2256, 10.1021/acs.chemrev.6b00021. 27078087

[3] C. A. Puckett and J. K. Barton , “Methods to Explore Cellular Uptake of Ruthenium Complexes,” Journal of the American Chemical Society 129 (2007): 46–47, 10.1021/ja0677564. 17199281 PMC2747593

[4] P. Spence , J. Fielden , and Z. A. E. Waller , “Beyond Solvent Exclusion: I‐Motif Detecting Capability and an Alternative DNA Light‐Switching Mechanism in a Ruthenium(II) Polypyridyl Complex,” Journal of the American Chemical Society 142 (2020): 13856–13866, 10.1021/jacs.0c04789. 32786817

[5] H. Lian , X. Cheng , H. Hao , et al., “Metal‐containing Organic Compounds for Memory and Data Storage Applications,” Chemical Society Reviews 51 (2022): 1926–1982, 10.1039/D0CS00569J. 35083990

[6] Q. Zhang , Y. Wang , C. Nickle , et al., “Molecular Switching by Proton‐Coupled Electron Transport Drives Giant Negative Differential Resistance,” Nature Communications 15 (2024): 8300, 10.1038/s41467-024-52496-y. PMC11436842 39333486

[7] S. Ohkoshi , S. Takano , K. Imoto , M. Yoshikiyo , A. Namai , and H. Tokoro , “90‐degree Optical Switching of Output Second‐Harmonic Light in Chiral Photomagnet,” Nature Photonics 8 (2013): 65–71, 10.1038/nphoton.2013.310.

[8] Y. Z. Yu , K. Y. Wong , and A. F. Garito , “Introduction to Nonlinear Optics,” Non‐linear Optics of Organic Molecules and Polymers (CRC Press, 1997).

[9] S. R. Marder , “Organic Nonlinear Optical Materials: Where We Have Been and Where We are Going,” Chemical Communications 2 (2006): 131–134, 10.1039/B512646K. 16372084

[10] M. G. Kuzyk , “Using Fundamental Principles to Understand and Optimize Nonlinear‐Optical Materials,” Journal of Materials Chemistry 19 (2009): 7444, 10.1039/b907364g.

[11] M. Pawlicki , H. A. Collins , R. G. Denning , and H. L. Anderson , “Two‐Photon Absorption and the Design of Two‐Photon Dyes,” Angewandte Chemie International Edition 48 (2009): 3244–3266, 10.1002/anie.200805257. 19370705

[12] S. R. Marder , J. W. Perry , and W. P. Schaefer , “Synthesis of Organic Salts With Large Second‐Order Optical Nonlinearities,” Science 245 (1989): 626–628, 10.1126/science.245.4918.626. 17837617

[13] B. J. Coe , J. A. Harris , I. Asselberghs , et al., “Quadratic Optical Nonlinearities of N ‐Methyl and N ‐Aryl Pyridinium Salts,” Advanced Functional Materials 13 (2003): 347–357, 10.1002/adfm.200300026.

[14] B. J. Coe , J. Fielden , S. P. Foxon , et al., “Syntheses and Properties of Two‐Dimensional, Dicationic Nonlinear Optical Chromophores Based on Pyrazinyl Cores,” Journal of Organic Chemistry 75 (2010): 8550–8563, 10.1021/jo101966r. 21080634

[15] S.‐H. Jang , J. Luo , N. M. Tucker , et al., “Pyrroline Chromophores for Electro‐Optics,” Chemistry of Materials 18 (2006): 2982–2988, 10.1021/cm052861i.

[16] B. J. Coe , J. Fielden , S. P. Foxon , et al., “Diquat Derivatives: Highly Active, Two‐Dimensional Nonlinear Optical Chromophores With Potential Redox Switchability,” Journal of the American Chemical Society 132 (2010): 10498–10512, 10.1021/ja103289a. 20617798

[17] B. J. Coe , J. Fielden , S. P. Foxon , et al., “Quadratic and Cubic Nonlinear Optical Properties of Salts of Diquat‐Based Chromophores With Diphenylamino Substituents,” Journal of Physical Chemistry A 114 (2010): 12028–12041, 10.1021/jp106473e. 20977249

[18] L. T. Cheng , W. Tam , S. H. Stevenson , G. R. Meredith , G. Rikken , and S. R. Marder , “Experimental Investigations of Organic Molecular Nonlinear Optical Polarizabilities. 1. Methods and Results on Benzene and Stilbene Derivatives,” Journal of Physical Chemistry 95 (1991): 10631–10643, 10.1021/j100179a026.

[19] H. Xu , F. Liu , D. L. Elder , et al., “Ultrahigh Electro‐Optic Coefficients, High Index of Refraction, and Long‐Term Stability From Diels–Alder Cross‐Linkable Binary Molecular Glasses,” Chemistry of Materials 32 (2020): 1408–1421, 10.1021/acs.chemmater.9b03725.

[20] H. Xu , L. E. Johnson , Y. de Coene , et al., “Bis(4‐dialkylaminophenyl)Heteroarylamino Donor Chromophores Exhibiting Exceptional Hyperpolarizabilities,” Journal of Materials Chemistry C 9 (2021): 2721–2728, 10.1039/D0TC05700B.

[21] H. Xu , D. L. Elder , L. E. Johnson , et al., “Electro‐Optic Activity in Excess of 1000 pm V^−1^ Achieved via Theory‐Guided Organic Chromophore Design,” Advanced Materials 33 (2021): 2104174, 10.1002/adma.202104174. 34545643

[22] G. Alcaraz , L. Euzenat , O. Mongin , et al., “Improved Transparency–Nonlinearity Trade‐off With Boroxine‐Based Octupolar Molecules,” Chemical Communications 22 (2003): 2766–2767, 10.1039/B308664J. 14651095

[23] Y. Shi , D. Frattarelli , N. Watanabe , et al., “Ultra‐High‐Response, Multiply Twisted Electro‐Optic Chromophores: Influence of π‐System Elongation and Interplanar Torsion on Hyperpolarizability,” Journal of the American Chemical Society 137 (2015): 12521–12538, 10.1021/jacs.5b04636. 26360110

[24] L. Beverina , A. Sanguineti , G. Battagliarin , et al., “UV Absorbing Zwitterionic Pyridinium‐Tetrazolate: Exceptional Transparency/Optical Nonlinearity Trade‐off,” Chemical Communications 47 (2011): 292–294, 10.1039/C0CC01652G. 20725681

[25] O. Maury and H. Le Bozec , “Molecular Engineering of Octupolar NLO Molecules and Materials Based on Bipyridyl Metal Complexes,” Accounts of Chemical Research 38 (2005): 691–704, 10.1021/ar020264l. 16171312

[26] H. Xiao , H. Yin , and X. Zhang , “Improved Nonlinearity–Transparency–Thermal Stability Trade‐Off With Spirobifluorene‐Bridged Donor‐π‐Acceptor Chromophores,” Organic Letters 14 (2012): 5282–5285, 10.1021/ol302443z. 23035950

[27] B. J. Coe , S. Houbrechts , I. Asselberghs , and A. Persoons , “Efficient, Reversible Redox‐Switching of Molecular First Hyperpolarizabilities in Ruthenium(II) Complexes Possessing Large Quadratic Optical Nonlinearities,” Angewandte Chemie International Edition 38 (1999): 366–369, 10.1002/(SICI)1521-3773(19990201)38:3<366::AID-ANIE366>3.0.CO;2-D. 29711640

[28] F. Paul , K. Costuas , I. Ledoux , et al., “Redox‐Switchable Second‐Order Molecular Polarizabilities With Electron‐Rich Iron σ‐Aryl Acetylides,” Organometallics 21 (2002): 5229–5235, 10.1021/om020303x.

[29] C. Sporer , I. Ratera , D. Ruiz‐Molina , et al., “A Molecular Multiproperty Switching Array Based on the Redox Behavior of a Ferrocenyl Polychlorotriphenylmethyl Radical,” Angewandte Chemie International Edition 43 (2004): 5266–5268, 10.1002/anie.200454150. 15455435

[30] P. Kaur , M. Kaur , G. Depotter , et al., “Thermally Stable Ferrocenyl “Push–pull” Chromophores With Tailorable and Switchable Second‐order Non‐linear Optical Response: Synthesis and Structure–Property Relationship,” Journal of Materials Chemistry 22 (2012): 10597, 10.1039/c2jm31387a.

[31] S. Di Bella , I. P. Oliveri , A. Colombo , C. Dragonetti , S. Righetto , and D. Roberto , Dalton Transactions 41 (2012): 7013. 22547326 10.1039/c2dt30702b

[32] E. Cariati , X. Liu , Y. Geng , et al., “Stimuli‐responsive NLO Properties of Tetrathiafulvalene‐Fused Donor–Acceptor Chromophores,” Physical Chemistry Chemical Physics 19 (2017): 22573–22579, 10.1039/C7CP04687A. 28809980

[33] I. Asselberghs , Y. Zhao , K. Clays , A. Persoons , A. Comito , and Y. Rubin , “Reversible Switching of Molecular Second‐Order Nonlinear Optical Polarizability Through Proton‐Transfer,” Chemical Physics Letters 364 (2002): 279–283, 10.1016/S0009-2614(02)01346-5.

[34] F. Castet , V. Rodriguez , J.‐L. Pozzo , L. Ducasse , A. Plaquet , and B. Champagne , “Design and Characterization of Molecular Nonlinear Optical Switches,” Accounts of Chemical Research 46 (2013): 2656–2665, 10.1021/ar4000955. 23865890

[35] J. Boixel , V. Guerchais , H. Le Bozec , et al., “Second‐Order NLO Switches From Molecules to Polymer Films Based on Photochromic Cyclometalated Platinum(II) Complexes,” Journal of the American Chemical Society 136 (2014): 5367–5375, 10.1021/ja4131615. 24635126

[36] P. Beaujean , F. Bondu , A. Plaquet , et al., “Oxazines: A New Class of Second‐Order Nonlinear Optical Switches,” Journal of the American Chemical Society 138 (2016): 5052–5062, 10.1021/jacs.5b13243. 26996994

[37] I. Asselberghs , K. Clays , A. Persoons , A. M. McDonagh , M. D. Ward , and J. A. McCleverty , “In Situ Reversible Electrochemical Switching of the Molecular First Hyperpolarizability,” Chemical Physics Letters 368 (2003): 408–411, 10.1016/S0009-2614(02)01890-0.

[38] L. Boubekeur‐Lecaque , B. J. Coe , K. Clays , S. Foerier , T. Verbiest , and I. Asselberghs , “Redox‐Switching of Nonlinear Optical Behavior in Langmuir−Blodgett Thin Films Containing a Ruthenium(II) Ammine Complex,” Journal of the American Chemical Society 130 (2008): 3286–3287, 10.1021/ja711170q. 18298121

[39] C. Karthika , S. R. Sarath Kumar , L. Kathuria , P. K. Das , and A. G. Samuelson , “In Situ Reversible Redox Switching of First Hyperpolarizability of Bimetallic Ruthenium Complexes,” Physical Chemistry Chemical Physics 21 (2019): 11079–11086, 10.1039/C9CP00946A. 31093630

[40] J. Quertinmont , P. Beaujean , J. Stiennon , et al., “Combining Benzazolo‐Oxazolidine Twins Toward Multi‐state Nonlinear Optical Switches,” Journal of Physical Chemistry B 125 (2021): 3918–3931, 10.1021/acs.jpcb.1c01962. 33851843

[41] M. Sadakane and E. Steckhan , “Electrochemical Properties of Polyoxometalates as Electrocatalysts,” Chemical Reviews 98 (1998): 219–238, 10.1021/cr960403a. 11851504

[42] O. Linnenberg , M. Moors , A. Notario‐Estévez , et al., “Addressing Multiple Resistive States of Polyoxovanadates: Conductivity as a Function of Individual Molecular Redox States,” Journal of the American Chemical Society 140 (2018): 16635–16640, 10.1021/jacs.8b08780. 30418764

[43] C. Busche , L. Vilà‐Nadal , J. Yan , et al., “Design and Fabrication of Memory Devices Based on Nanoscale Polyoxometalate Clusters,” Nature 515 (2014): 545–549, 10.1038/nature13951. 25409147

[44] S. Liu , D. G. Kurth , H. Möhwald , and D. Volkmer , “A Thin‐Film Electrochromic Device Based on a Polyoxometalate Cluster,” Advanced Materials 14 (2002): 225–228, 10.1002/1521-4095(20020205)14:3<225::AID-ADMA225>3.0.CO;2-F.

[45] B. R. Hood , Y. de Coene , C. F. Jones , et al., “Donor and Geometry Optimization: Fresh Perspectives for the Design of Polyoxometalate Charge Transfer Chromophores,” Inorganic Chemistry 64 (2025): 8408–8420, 10.1021/acs.inorgchem.5c00915. 40228151 PMC12042256

[46] B. R. Hood , Y. de Coene , A. V. Torre Do Vale Froes , et al., “Electrochemically‐Switched 2nd Order Non‐Linear Optical Response in an Arylimido‐Polyoxometalate With High Contrast and Cyclability,” Angewandte Chemie International Edition 62 (2023): e202215537, 10.1002/anie.202215537. 36448963 PMC10107823

[47] A. Al‐Yasari , N. Van Steerteghem , H. El Moll , K. Clays , and J. Fielden , “Donor–Acceptor Organo‐Imido Polyoxometalates: High Transparency, High Activity Redox‐active NLO Chromophores,” Dalton Transactions 45 (2016): 2818–2822, 10.1039/C6DT00115G. 26815652

[48] A. Al‐Yasari , N. Van Steerteghem , H. Kearns , et al., “Organoimido‐Polyoxometalate Nonlinear Optical Chromophores: A Structural, Spectroscopic, and Computational Study,” Inorganic Chemistry 56 (2017): 10181–10194, 10.1021/acs.inorgchem.7b00708. 28809116

[49] A. Al‐Yasari , P. Spence , H. El Moll , et al., “Fine‐tuning Polyoxometalate Non‐linear Optical Chromophores: A Molecular Electronic “Goldilocks” Effect,” Dalton Transactions 47 (2018): 10415–10419, 10.1039/C8DT01491D. 29947391

[50] C. F. Jones , B. R. Hood , Y. de Coene , et al., “Bridge Improvement Work: Maximising Non‐Linear Optical Performance in Polyoxometalate Derivatives,” Chemical Communications 60 (2024): 1731. 38240142 10.1039/d3cc05433k

[51] T. Zhang , X.‐H. Wei , Y. Zuo , and T.‐Y. Ma , “Switchable NLO Response Induced by Redox Properties of Keggin‐type Polyoxometalate Hybrids [PX_11_O_39_{Sn(p‐C_6_H_4_I)}]^4−^ (X = Mo, W),” Journal of Molecular Structure 1217 (2020): 128364, 10.1016/j.molstruc.2020.128364.

[52] V. S. Lin and M. J. Therien , “The Role of Porphyrin‐to‐Porphyrin Linkage Topology in the Extensive Modulation of the Absorptive and Emissive Properties of a Series of Ethynyl‐ and Butadiynyl‐Bridged Bis‐ and Tris(porphinato)Zinc Chromophores,” Chemistry – A European Journal 1 (1995): 645–651, 10.1002/chem.19950010913.

[53] S. M. LeCours , S. G. DiMagno , and M. J. Therien , “Exceptional Electronic Modulation of Porphyrins Through Meso ‐Arylethynyl Groups. Electronic Spectroscopy, Electronic Structure, and Electrochemistry of [5,15‐Bis[(aryl)ethynyl]‐ 10,20‐diphenylporphinato]Zinc(II) Complexes. X‐ray Crystal Structures of [5,15‐Bis[(4‐fluorophenyl)ethynyl]‐ 10,20‐diphenylporphinato]Zinc(II) and 5,15‐Bis[(4‐methoxyphenyl)ethynyl]‐10,20‐diphenylporphyrin,” Journal of the American Chemical Society 118 (1996): 11854–11864, 10.1021/ja962403y.

[54] N. P. Kamat , Z. Liao , L. E. Moses , et al., “Sensing Membrane Stress With Near IR‐Emissive Porphyrins,” PNAS 108 (2011): 13984–13989, 10.1073/pnas.1102125108. 21844376 PMC3161589

[55] R. E. Sioda , D. O. Cowan , and W. S. Koski , “Study of the Radical Anion Formation of some Diphenylacetylenes in Dimethylformamide,” Journal of the American Chemical Society 89 (1967): 230–234, 10.1021/ja00978a008.

[56] M. Fournier and J. P. Launay , “The Analog of Surface Molybdenyl Ion in Mo/SiO2 Supported Catalysts: The Isopolyanion Mo6O193− Studied by EPR and UV‐Visible Spectroscopy. Comparison With Other Molybdenyl Compounds,” Journal of Chemical Physics 71 (1979): 1954.

[57] C. Sanchez , J. Livage , J. P. Launay , M. Fournier , and Y. Jeannin , “Electron Delocalization in Mixed‐Valence Molybdenum Polyanions,” Journal of the American Chemical Society 104 (1982): 3194–3202, 10.1021/ja00375a044.

[58] E. Rtibi , M. Abderrabba , S. Ayadi , and B. Champagne , “Theoretical Assessment of the Second‐Order Nonlinear Optical Responses of Lindqvist‐Type Organoimido Polyoxometalates,” Inorganic Chemistry 58 (2019): 11210–11219, 10.1021/acs.inorgchem.9b01857. 31390191

[59] J. L. Oudar and D. S. Chemla , “Hyperpolarizabilities of the Nitroanilines and Their Relations to the Excited State Dipole Moment,” Journal of Chemical Physics 66 (1977): 2664–2668, 10.1063/1.434213.

[60] J. L. Oudar , “Optical Nonlinearities of Conjugated Molecules. Stilbene Derivatives and Highly Polar Aromatic Compounds,” Journal of Chemical Physics 67 (1977): 446–457, 10.1063/1.434888.

[61] Y. Zhang , F. Castet , and B. Champagne , “Theoretical Investigation of the First Hyperpolarizability Redox‐Switching in a Ruthenium Complex,” Chemical Physics Letters 574 (2013): 42–46, 10.1016/j.cplett.2013.04.071.

