# Supplementary material for: Electrochemical Switch‐On of Nonlinear Optical Response in a Nitro‐Functionalized Arylimido‐Polyoxometalate
Source: Angew Chem Int Ed Engl. 2026 Jun 30;65(34):e4949539. doi: 10.1002/anie.4949539 (PMC13480563; doi:10.1002/anie.4949539)
Supplement: Supplementary file 1 [file ANIE-65-e4949539-s002.pdf]

Supplementary information for:

Electrochemical Switch-On of Non-Linear Optical Response in a Nitro-Functionalized Arylimido-Polyoxometalate

Claire F. Jones, Bethany R. Hood, Yovan de Coene, Hayleigh Kearns, Karen Faulds, Benoît Champagne, Koen Clays and John Fielden

1. Synthetic and General Methods

Materials and Procedures. Acetonitrile (MeCN) for synthesis and electrochemistry was freshly distilled over CaH_2 under a nitrogen atomsophere,[1] or dried using an MBraun SPS-800 solvent purification system with water content determined by coulometric Karl Fisher titration using a Metrohm 899 coulometer before use. Dry dimethyl sulfoxide (DMSO) was purchased from Sigma Aldrich (SureSeal) and Acros Organics (Acro Seal). Acetonitirile for HRS switching studies was purchased dry from Sigma Aldrich in SureSeal bottles and used as received. All other reagents and solvents were obtained as ACS grade from Sigma Aldrich, Alfa Aesar, Fisher Scientific, Fluorochem, Acros Organics, or Apollo Scientific and used as supplied. Deuterated solvents were obtained from Eurisotop, Cambridge Isotope Laboratories, or Acros Organics and used as supplied. Tetrabutylammonium hexamolybdate,[2] 4-iodo-2,6-diisopropylaniline (**P1**),[3,4] and its polyoxometalate-imido derivative $[NBu_4]_2$[**P2**][4] were synthesized according to previously published methods. The Sonogashira coupling reaction was adapted from known procedures,[4-7] and performed under an atmosphere of dry argon using standard Schlenk techniques. Unless otherwise stated, preparation of organic precursors was carried out in air.

General Physical Measurements. FT-IR spectra were measured using a Bruker FT-IR XSA spectrometer. ^{1}H- and ^{13}C-NMR spectra were acquired using a Bruker Ascend 500 (500 MHz) spectrometer and all shifts are quoted with respect to TMS using the solvent signals as secondary standard (s = singlet, d = doublet, t = triplet, q = quartet, quin = quintet, sex = sextet, *a*sex = apparent sextet, hept = heptet, dt = doublet of triplets, m = multiplet). Some quaternary carbon signals were not observed, similar to previously synthesised arylimido-polyoxometalates.[6-8] Elemental analyses and accurate mass spectrometry were outsourced to the University of Manchester, and the John Innes Centre (Norwich) respectively. UV-Vis spectra were obtained by using an Agilent Cary 60 UV-Vis spectrophotometer. UV-Vis NIR spectra were acquired with an Agilent Cary 5000 spectrophotometer.

Synthesis of Polyoxometalate Derivatives and Precursors. The synthetic route to $[NBu_4]_2$[**1**] is illustrated in Scheme S1, and the method for the Sonogashira coupling to form $[NBu_4]_2$[**1**] from $[NBu_4]_2$[**P2**] is fully described below.

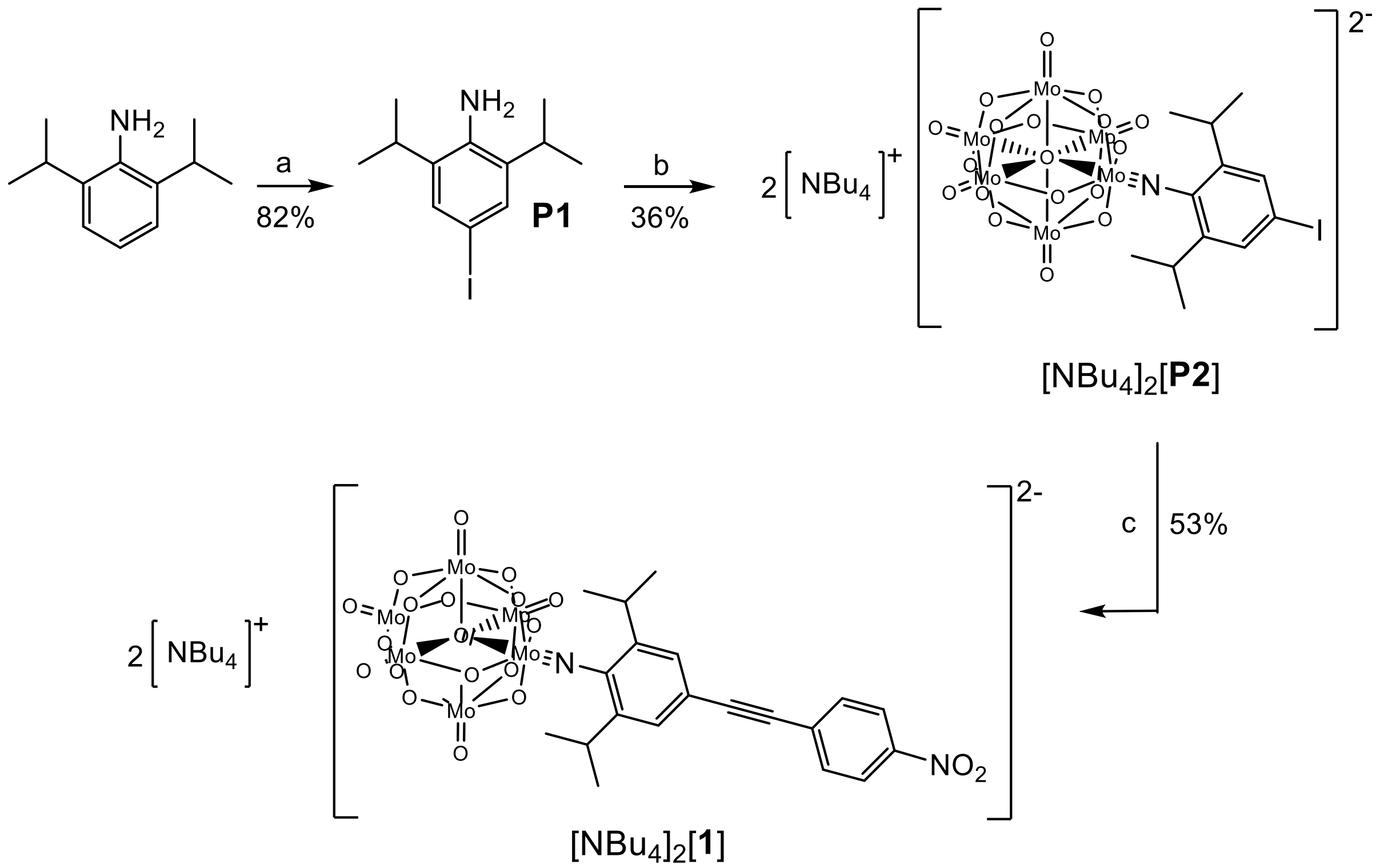

Scheme S1 Synthetic route to compound $[NBu_4]_2$[**1**]. Reagents and conditions: (a) ICl, $NaHCO_3$, rt, 18h.[3,4] (b) $[NBu_4]_2[Mo_6O_{19}]$, DCC, DMSO, 70 °C, 10 h.[4] (c) 1-ethynyl-4-nitrobenzene, CuI, $PdCl_2$ (PPh3)2, Et_3N, MeCN, rt, 0.5 h.

Preparation of $[NBu_4]_2[Mo_6O_{18}(C_{20}H_{20}N_2O_2)]$ ($[NBu_4]_2$[1])

Compound **P2** (0.309 g, 0.19 mmol), 1-ethynyl-4-nitrobenzene (0.033 g, 0.22 mmol), CuI (0.002 g, 0.01 mmol), *bis*(triphenylphosphine)palladium(II) chloride (0.038 g, 0.05 mmol) and K_2CO_3 (0.180 g, 1.30 mmol) were degassed then dissolved in 7.5 mL of dry acetonitrile before dry triethylamine (0.4 ml, 2.87 mmol) was added. The resulting mixture was stirred at room temperature for 30 minutes before filtering, evaporating *in vacuo* to 1 mL, and pouring into 50 mL of ethanol. The resulting orange precipitate was collected by filtration, washed with ethanol and diethyl ether and then dried in vacuo to give crude product as an orange solid (0.167 g, 0.10 mmol) with a 53% yield. Further purification of 145 mg of sample was achieved by crystallisation from hot acetonitrile to give pure $[NBu_4]_2$[1] (47 mg, 0.028 mmol) in an overall 17% yield. ^{1}H-NMR (500 MHz, CD_3CN): δ 8.24 (d, J = 9.0 Hz, 2H), 7.74 (d, J = 9.0 Hz, 2H), 7.40 (s, 2H), 3.86 (hept, J = 6.9 Hz, 2H), 3.10 – 3.07 (m, 16H), 1.63 – 1.57 (m, 16H), 1.36 (*a*sex, J = 7.5 Hz, 16H), 1.32 (d, J = 6.8 Hz, 12H), 0.97 (t, J = 7.4 Hz, 24H). ^{13}C-NMR (126 MHz, CD_3CN) δ 149.86, 133.37, 126.8, 124.73, 59.27, 29.47, 24.28, 23.89, 20.28, 13.76, 9.21. Anal (Calcd) % for $C_{52}H_{92}N_4O_{20}Mo_6$: C 37.42 (37.67), H 5.57 (5.62), N, 3.36 (3.29). HRMS (ESI, MeCN) = calcd for $C_{20}H_{20}N_2Mo_6O_{20}^{2-}$ ([**1**]$^{2-}$) 591.7489, found 591.7443. FTIR (ATR): 2961 (m), 2934 (sh), 2873(m), 2199 (m), 1594 (m), 1516 (m), 1461 (m), 1380 (m), 1363 (s), 1339 (m), 1245 (w), 1172 (w), 1153 (w), 1107 (w), 974 (m), 942 (vs) 882 (m), 854 (m) 767 (vs), UV-vis (MeCN) λ, nm (ε, M^{-1} cm^{-1}): 200 (67.7×10^3); 246 (37.3×10^3); 276 (30.4×10^3); 399 (53.7×10^3).

2. X-ray Crystallographic Details

Sample Growth, Data Collection and Refinement. Crystals of $[NBu_4]_2$[**1**] were grown by diffusion of diethyl ether into an acetonitrile solution. Data was collected on a Rigaku XtalLab Synergy S diffractometer using a Photon-Jet Cu or Mo microfocus source and Hypix hybrid photon counting detector. Data reduction, cell refinement and absorption correction were carried out using Rigaku CrysAlisPro,[9] and the structure was solved with SHELXT[10] in Olex2 V1.5.[11] Refinement was achieved by full-matrix least-squares on all F_0^2 data using SHELXL (v. 2018-3),[12] also in Olex 2 V1.5. The asymmetric unit (Figures S1) contains the complete molecular anion and both tetrabutylammonium cations. X-ray crystallographic data and refinement parameters are summarised in Table S1.

Table S1. Crystallographic Data and Refinement Details for $[NBu_4]_2$[**1**]

Formula	$C_{52}H_{92}Mo_6N_4O_{20}$
M	1668.93
crystal system	Triclinic
space group	*P*-1
a/Å	11.9929(3)
b/Å	12.7474(3)
c/Å	22.9776(5)
α/deg	85.427(2)
β/deg	75.303(2)
γ/deg	78.089(2)
V/Å^3	3323.30(14)
Z	2
Radiation	Mo Kα (λ = 0.71073 Å)
T/K	100.00(11)
μ/mm^{-1}	1.167
Cryst. size/mm	0.12 × 0.1 × 0.08
Cryst. description	Orange plate
No. reflns collected	99719
No. of indep. reflns (R_{int})	17951 [R(int) = 0.0500]
$2\theta_{full}$/deg (completeness)<br>$2\theta_{max}$/deg (completeness)	25.242 (99.9%)<br>30.953 (84.9%)
Reflections with $I > 2\sigma(I)$	14421
Goodness-of-fit on F^2	1.068
final R_1, wR_2 $[I > 2\sigma(I)]^a$	R1 = 0.0359, wR2 = 0.0886
(all data)	R1 = 0.0482, wR2 = 0.0934
Peak and hole/e Å^{-3}	1.33 and -0.86

Structures and Selected Bond Lengths. A representation of the asymmetric unit of $[NBu_4]_2$[**1**] is shown in Figure S1. Selected bond lengths and angles for the anion $[\mathbf{1}]^{2-}$ are collected in Tables S2, together with those of the isopropyl-free analogue $[\mathbf{1H}]^{2-}$.[7] Both structures show the typical imido-Lindqvist pattern of a shortened bond length from the imido-Mo (Mo^{im}) to the central oxygen (O^c), lengthened equatorial bond lengths from Mo^{im} to the oxygens bridging to the belt Mo positions (Mo^b), and a lengthened axial bond length from the *trans*-Mo (Mo^t) to O^c.[6-8] There is no pattern in the terminal Mo=O distances which are typically in the range of 1.65 to 1.70 Å. The main difference between the structures is the slightly longer and much more linear Mo-N-C bond angles in $[\mathbf{1}]^{2-}$ forced by the isopropyl groups. Bond distances within the imido-aryl ligands of $[\mathbf{1}]^{2-}$ do not show any quinoidal character. Twist around the alkyne bond is decreased in $[\mathbf{1}]^{2-}$ compared to $[\mathbf{1H}]^{2}$, suggesting strengthened electronic conjugation.

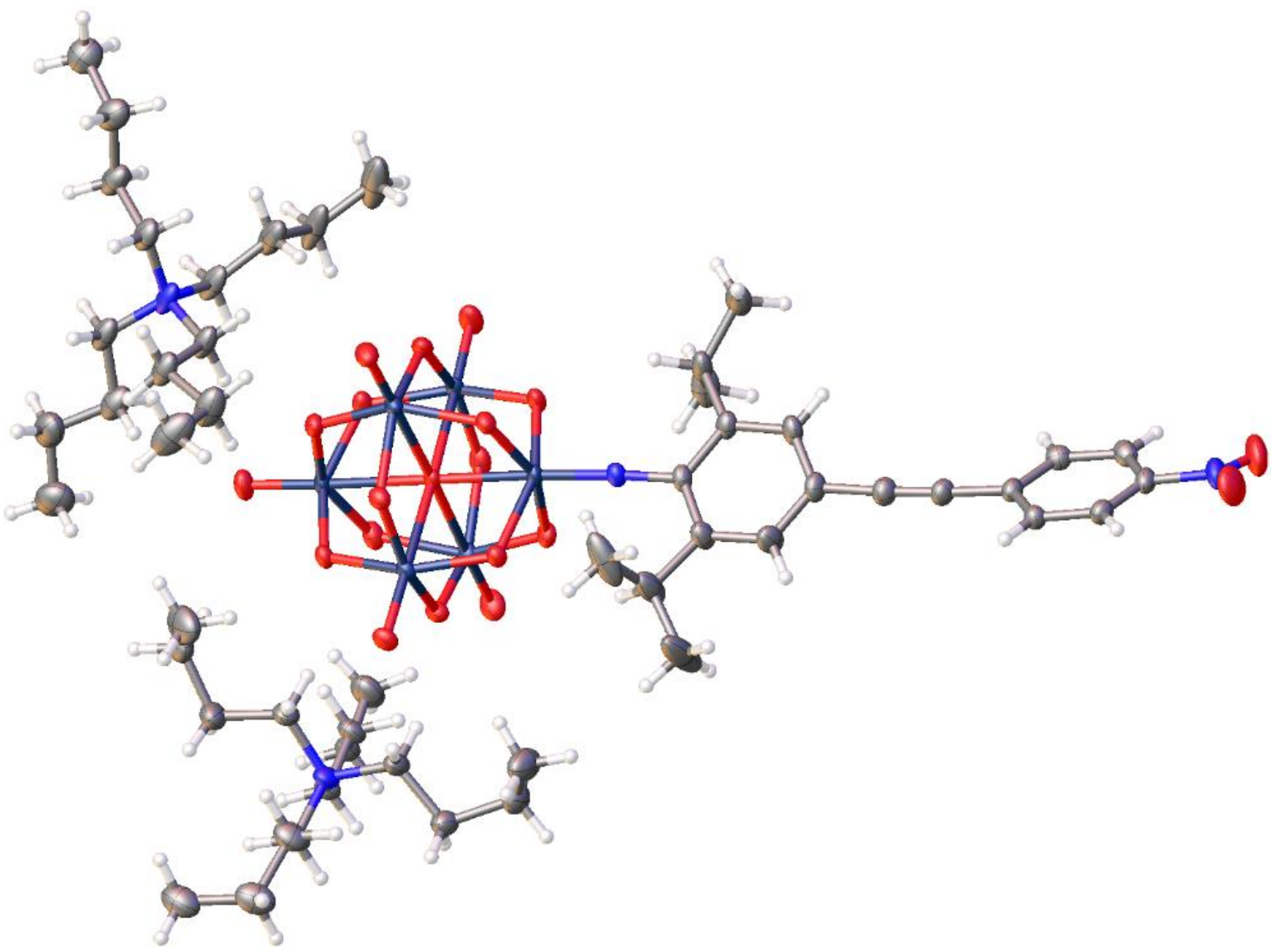

Figure S1 ORTEP representation of the asymmetric unit in $[NBu_4]_2$[**1**]. Thermal ellipsoids are at the 30% probability level and disordered parts are omitted for clarity. Colour scheme: Mo is navy; O, red; C, gray; N, blue; H atoms are represented by white spheres of arbitrary radii.

Table S2 Selected bond lengths (Å) and angles (°) of the $\{Mo_6\}$ units in anions $[\mathbf{1}]^{2-}$ and $[\mathbf{1H}]^{2-}$

	Mo^{im}-N	Mo^{im}-O^{c}	Mo^{im}-O^{b} (average)	Mo^{t} -O^{b} (average)	Mo^{t}-O^{c}	N-C	Mo^{im}-N-C	Alkyne torsion
$[\mathbf{1}]^{2-}$	1.742(2)	2.202(2)	1.952(2)	1.947 (2)	2.364(2)	1.385(3)	176.0(2)	10.44
$[\mathbf{1H}]^{2-}$ [a]	1.748 (6)	2.190 (5)	1.966 (5)	1.934 (5)	2.350 (5)	1.381 (9)	163.46 (6)	16.63

[a] Previously published in ref. 7

3. Raman Spectroscopy

POM derivative $[NBu_4]_2$[**1**] was prepared as a 5 mM solution in acetonitrile and analyzed in a glass cuvette using 600 µL of the solution. Raman measurements were performed using Snowy Range (SnRI) portable Raman spectrometers. Two excitation sources were used; a diode laser with 532 nm excitation (50 mW of power) and a diode laser with 785 nm excitation (100 mW). Acquisition times were 0.2 s at 532 nm and 1 s at 785 nm. The SnRI 532 nm spectrometer has a wavenumber range from 200 to 3200 cm^{-1} whereas the 785 nm spectrometer has a range from 200 to 2000 cm^{-1}. The Raman measurements were normalized to an ethanol standard with respect to the peak at 881 cm^{-1}. Spectra were baseline corrected using asymmetric least-squares in MATLAB software (version R2019a).

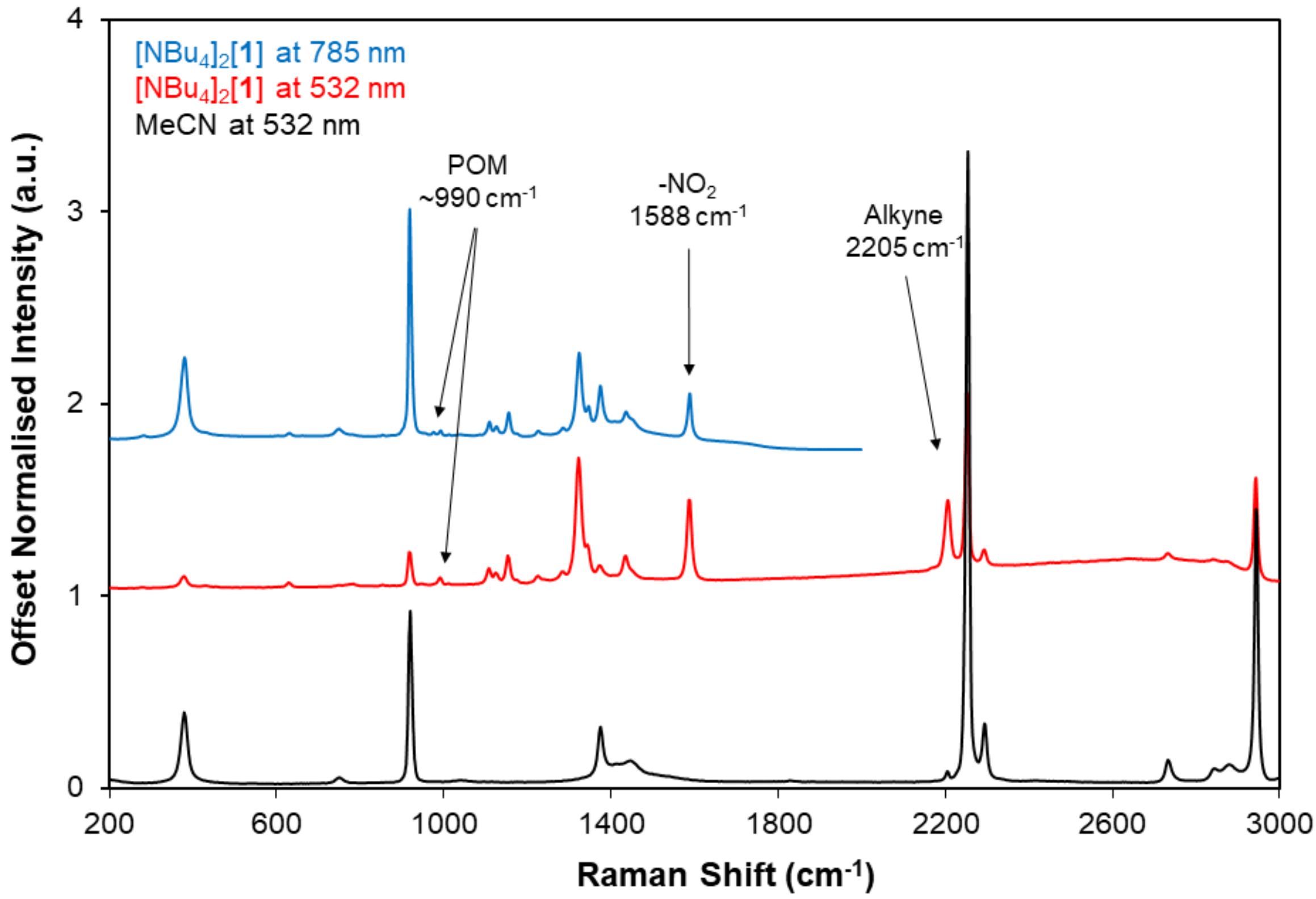

Figure S2 Raman spectra of $[NBu_4]_2[Mo_6O_{18}N(^iPr)_2PhCCPhNO_2]$ ($[NBu_4]_2[\mathbf{1}]$ excited at 532 (red spectrum) and 785 nm (blue spectrum), plus a reference spectrum of MeCN only (black spectrum) excited at 532 nm. This is consistent with weak resonance enhancement by the tail of the visible absorption band, and participation of both POM and $-NO_2$ in the low energy CT transitions.

4. Electrochemistry

Cyclic Voltammetry. All measurements were carried out using Autolab PGStat 302 potentiostat/galvanostat. A conventional three-electrode cell was used with an Ag wire pseudo reference electrode, glassy carbon working electrode and Pt wire auxiliary electrode. Acetonitrile was freshly distilled (from CaH_2) and $[N(C_4H_9\text{-}n)_4]BF_4$,[13] was used as the supporting electrolyte. Solutions containing *ca*. 0.8 mM analyte (0.1 M electrolyte) were degassed by purging with argon and blanketed with a continuous flow of argon throughout the experiments. $E_{1/2}$ values were calculated from $(E_{pa} + E_{pc})/2$ at a scan rate of 100 mV s^{-1} and referenced to Fc/Fc^+. Methods used to compute potentials by DFT are described in Section 6.

Bulk Electrolysis. Bulk electrolysis was performed in a three compartment cell with counter and reference electrodes segregated from the working compartment by sintered glass disks to minimize potential drift and prevent re-oxidation of analyte. A Pt gauze working electrode was used, with Ag wire reference and Pt counter and vigorous stirring was applied. An additional glassy carbon working electrode was used to measure cyclic voltammograms before and after reduction. Acetonitrile was freshly distilled (from CaH_2) and $[N(C_4H_9\text{-}n)_4]BF_4$,[13] was used as the supporting electrolyte. Solutions containing ca. 0.8 mM analyte (0.1 M electrolyte) were degassed by purging with argon and blanketed with a continuous flow of argon throughout the experiments. Reduction of the bulk sample was achieved by application of a potential of -0.7

V vs $Ag^{0/+}$ to the vigorously stirring solution for a total of 23 minutes. After this time the current had plateaued at a current of near 0 A showing no further reduction was taking place.

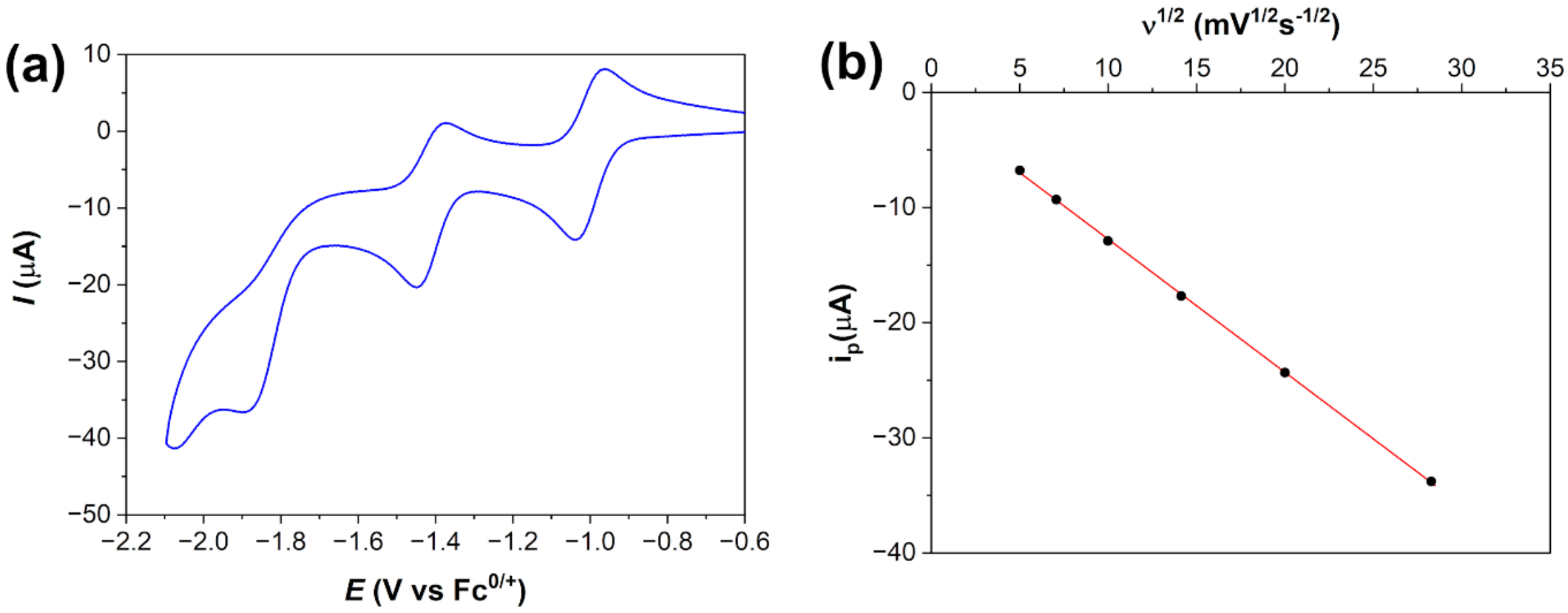

Figure S3 (a) Full range cyclic voltammograms of $[NBu_4]_2$[**1**]. Scan rate 100 mV s^{-1}, GC working electrode, 0.1 M $[NBu_4][BF_4]$ in acetonitrile as electrolyte. **(b)** Peak current *vs* square root of scan rate for the first POM reduction of $[NBu_4]_2$[**1**].

Table S3 Experimental and computed redox potentials for $[NBu_4]_2$[**1**] in acetonitrile

$E_{1/2}$ [**1**]$^{2-/3-}$ / V *vs* Fc/Fc^+		$E_{1/2}$ [**1**]$^{3-/4-}$ / V *vs* Fc/Fc^+			E_{pc} [**1**]$^{4-/5-}$ (irrev)[a] / V *vs* Fc/Fc^+
Expt (ΔE_p / mV)[b]	Calcd[c]	Expt (ΔE_p / mV)[b]	Calcd[c] (S=1)	Calcd[c] (S=0)	Expt[b]
-1.00 (73)	-0.81	-1.41 (75)	-1.94	-1.97	-1.90

[a] Larger peak current and irreversibility imply this is not a simple one-electron process, for this reason no attempt has been made to compute potentials. [b] Conditions: *ca.* 10^{-3} M in analyte, 0.1 M $[NBu_4][BF_4]$ in MeCN at a glassy carbon working electrode, scan rate 100 mV s^{-1}. [c] Calculated from structures obtained using the IEFPCM(solvent = acetonitrile) TD-DFT/ωB97X-D/6-311G(d)/LanL2TZ level of approximation, see computational section for details. The difference between computed and experimental values is because computed values do not take account of ion pairing, or explicit interactions with solvent.

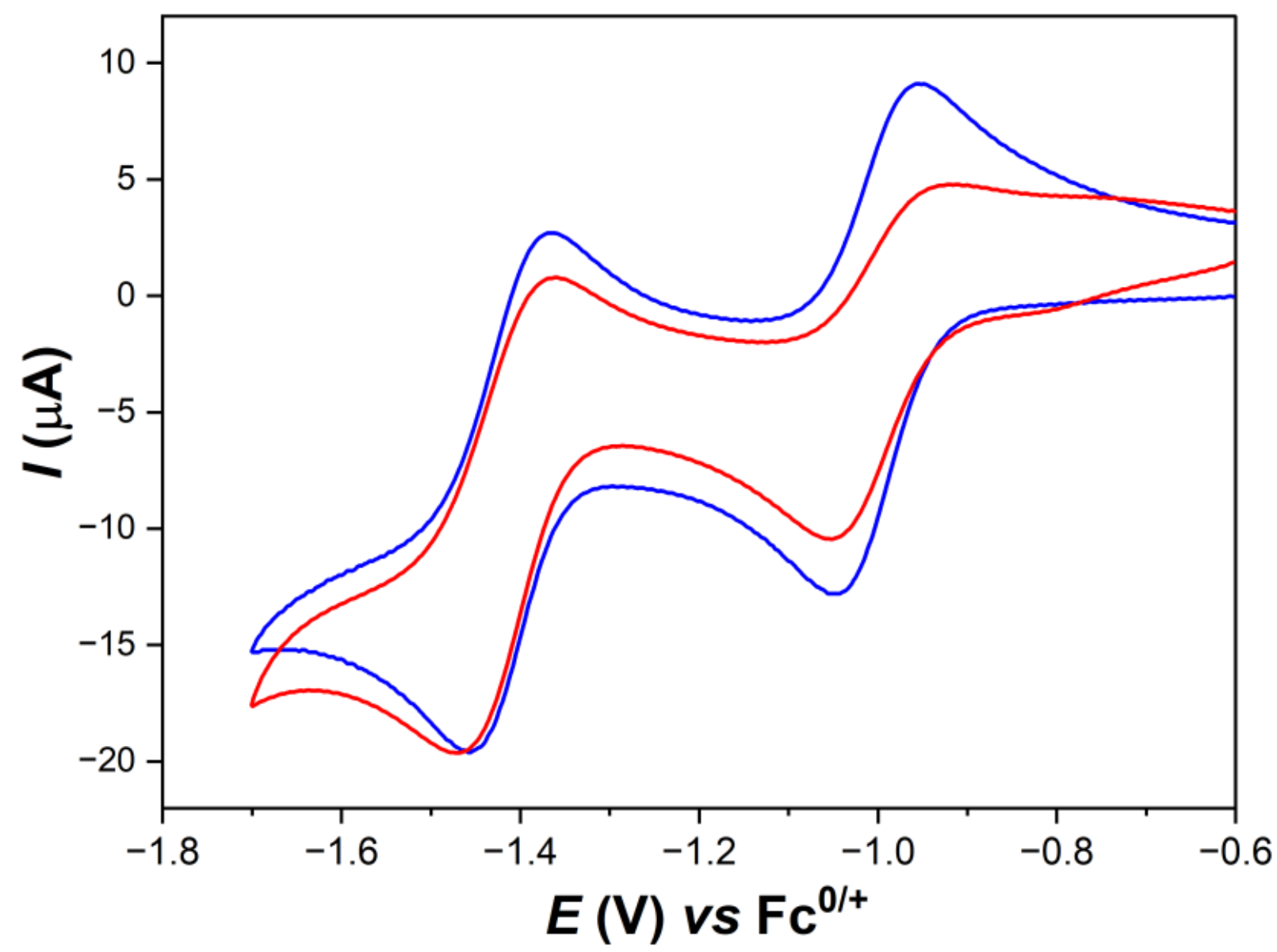

Figure S4 Cyclic voltammogram of $[NBu_4]_2$[**1**] and after bulk electrolysis for 23 min at -0.7 V vs Ag/Ag^+ (*ca.* -1.1 V *vs* $Fc^{0/+}$). To aid comparison, the baseline current of the post-BE scans at the start potential have been shifted to the same value as the initial scan.

5. UV-visible and Electrochemical Data for ⁱPr-free Analogue $[1H]^{2-}$ vs $[1]^{2-}$

Data for ⁱPr-free [**1H**]$^{2-}$ were previously published,[7] and are presented here with the data for [**1**]$^{2-}$ for convenient comparison. Only the reversible {Mo_6} reduction is shown, as electrochemical study of [**1H**]$^{2-}$ did not extend beyond this.

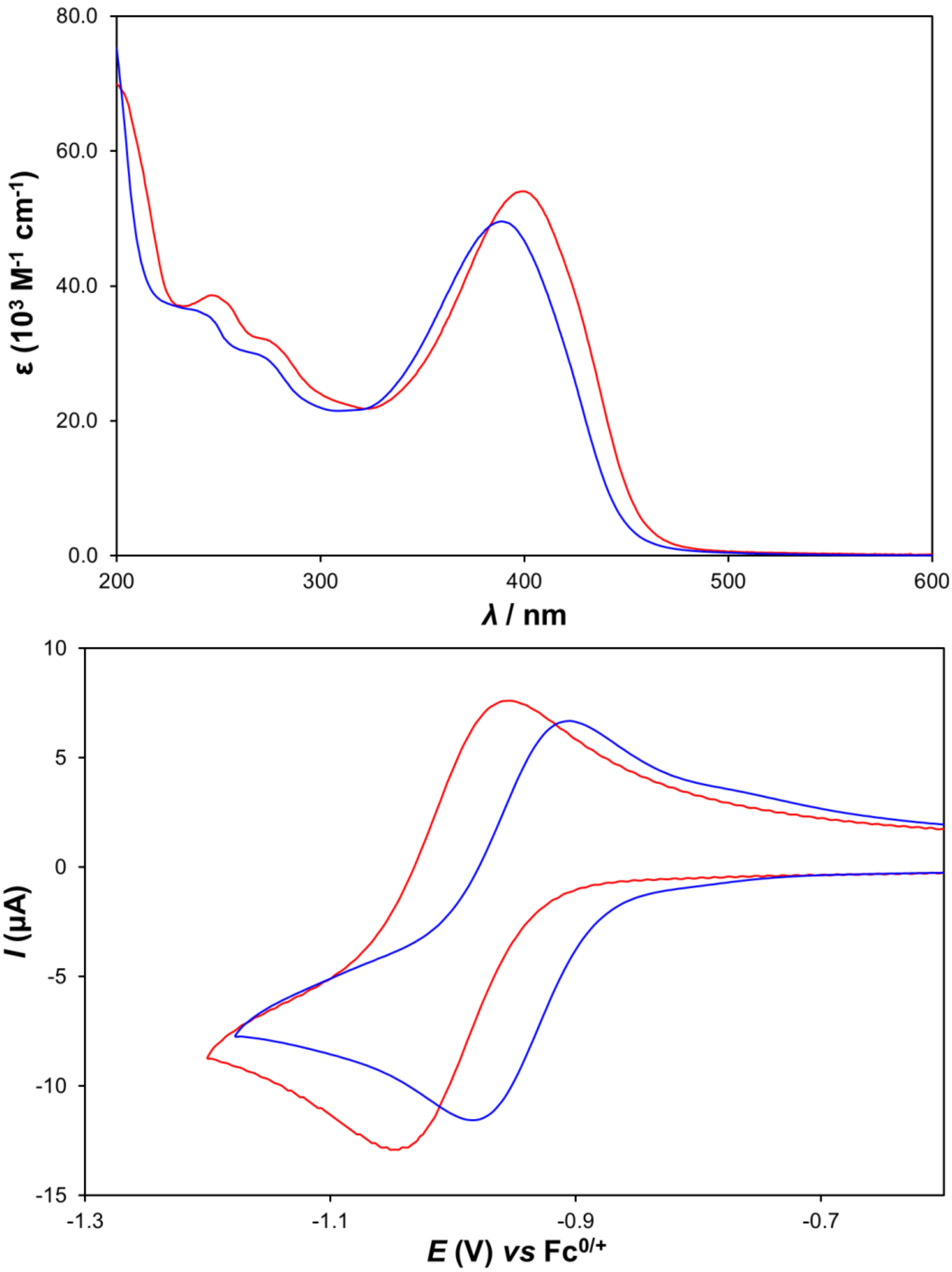

Figure S5 (a) UV-vis spectra of $[NBu_4]_2$[**1**] (red) and $[NBu_4]_2$[**1H**] (blue) obtained in acetonitrile at room temperature. **(b)** Cyclic voltammograms of $[NBu_4]_2$[**1**] (red) and $[NBu_4]_2$[**1H**] (red) showing the reversible POM reduction peak. Scan rate 100 mV s^{-1}, GC working electrode, 0.1 M $[NBu_4][BF_4]$ in

acetonitrile as electrolyte. $[NBu_4]_2$[**1H**] was previously published.[7] The small increase in current seen for $[NBu_4]_2$[**1H**] around -0.8 V is due to contamination with a small amount of $[Mo_6O_{19}]^{2-}$, which can be extremely difficult to remove.

Table S4 UV-vis Absorption and Electrochemical data for $[\mathbf{1}]^{2-}$ and $[\mathbf{1H}]^{2-}$ in acetonitrile

Anion **X**	λ_{max} / nm (ε, 10^3 M^{-1} cm^{-1})[a]	E_{max} / eV	UV-vis assignment	$[\mathbf{X}]^{2-/3-}$ $E_{1/2}$ / V *vs* Fc/Fc$^+$ (ΔE_p / mV) [b]
$[\mathbf{1}]^{2}$	246 (37.3)<br>276 (30.4)<br>399 (53.7)	5.04<br>4.49<br>3.11	O→Mo and π→π*<br>O→Mo and π→π*<br>LPCT[c] and π→NO_2	-1.001 (73)
$[\mathbf{1H}]^{2-}$ [d]	241 (36.0)<br>269 (29.9)<br>389 (49.6)	5.08<br>4.37<br>3.02	O→Mo and π→π*<br>O→Mo and π→π*<br>LPCT and π→NO_2	-0.986 (64)

[a] Obtained at *ca.* 10^{-5} M in MeCN. [b] *ca.* 10^{-3} M in analyte, 0.1 M $[NBu_4][BF_4]$ in MeCN at a glassy carbon working electrode, scan rate 100 mV s^{-1}. [c]Aryl-imido ligand to polyoxometalate charge transfer. [d] Data previously reported in Ref. 7.

5. Quantum chemistry calculations

Geometry optimizations. Full geometry optimizations were performed at the density functional theory (DFT) level using the ωB97X-D exchange-correlation functional (XCF).[14] This range-separated hybrid functional combines a modified B97 exchange functional[15] for short-range interactions (from 78 to 0%) with HF exchange for long-range interactions (from 22 to 100%), using the default range-separating parameter, ω = 0.2 $Bohr^{-1}$. Correlation is described by the B97 correlation functional,[15] and empirical atom-atom London dispersion corrections are included as well. For the C, H, N, and O atoms, the atomic basis set consists of 6-311G(d)[16] while LANL2TZ[17] is used for the Mo atoms. TIGHT convergence thresholds on the residual forces on the atoms (1.5 x 10^{-5} Hartree/Bohr or Hartree/radian) were applied. The reliability of the ωB97X-D/6-311G(d)/LANL2TZ method for the geometry optimization of POM derivatives was demonstrated in comparison with other XC functionals in a previous work.[18] To describe the solvent effects (acetonitrile), geometry optimizations were performed in solution using the integral equation formalism (IEF) of the polarizable continuum model (PCM) (IEF-PCM), which represents the solvent by a dielectric continuum characterized by its dielectric permittivity (ε_0 = 35.688 for acetonitrile).[19] For the reduced state $[\mathbf{1}]^{3-}$, unrestricted settings were used, for the ground state the values of the $\hat{S}^2$ operator was $\cong 0.75$, indicating neglible spin contamination.

Properties of the excited states. Using the ground state optimized geometries, the time-dependent density functional theory (TD-DFT) method[20] with the same XCF, basis set, and IEF-PCM scheme was used to characterize the (lowest-energy) excited states, considering their vertical excitation energies, $\Delta E_{ge} = E_e - E_g$ (from the ground state g to the excited state e), the corresponding transition dipole moment, μ_{ge}, which is related to the oscillator strength, $f_{ge} = \frac{2}{3}\, \Delta E_{ge}\, \mu_{ge}^2$, and the difference of dipole moment between states g and e, $\Delta\mu_{ge} = \mu_e - \mu_g$. The excited state dipole moments were calculated by using the nonequilibrium IEF-PCM solvation approach (ε_∞ = 1.807 for acetonitrile). Nonequilibrium solvation TD-DFT calculations also provided the difference of electron density between the excited and the ground states, $\Delta\rho(\vec{r}) = \rho_e(\vec{r}) - \rho_g(\vec{r})$, and the change in dipole moment between the ground and excited states, $\Delta\mu_{ge}$. The 300 lowest excitation energies (and oscillator strengths) were

calculated. For the reduced species, unrestricted calculations were carried out. For the ground state, the value of the $\hat{S}^2$ operator was ≅0.75, indicating negligible spin contamination. Small spin contaminations were observed for several excited states, and values of $\hat{S}^2$are presented in Table S4.

First hyperpolarizability. Using again the same optimized geometries, the SHG β tensor components were evaluated by employing the quadratic response TD-DFT method[22,23] with the same basis set, XCF, and solvation model as for the excited states calculations. Both static and dynamic (incident wavelengths of 1064 nm and 1500 nm) responses were calculated. Computing β of large compounds, including those having donor and/or acceptor substituents, is a challenge for TD-DFT because of the intrinsic nonlocal nature of the response and the approximate XCFs (potential and kernel).[24] However, ωB97X-D is a reliable XCF for calculating the β tensors owing to its substantial amount of long-range HF exchange, as demonstrated in previous investigations where the performance of DFT XCFs was assessed with respect to benchmark wavefunction methods.[25-27] Ref. 19 has also evidenced that using local XCFs or XCFs with small amount of HF exchange leads to overestimated first hyperpolarizabilities. The unit sphere representation (USR) was adopted[28] to visualize the β tensors. First, the second-order induced electric dipole moments

$$\vec{\mu}_{ind} = \vec{\vec{\beta}}: \vec{E}^2(\theta, \phi) \quad (1)$$

are evaluated, where $\vec{\vec{\beta}}$ is the first hyperpolarizability tensor and $\vec{E}(\theta, \phi)$ is a unit vector of electric field, of which the polarization is defined by the θ and ϕ angles (spherical coordinates). Then, the induced dipoles are plotted on a sphere centered on the molecule center of mass. This allows highlighting the directions along which the second-order polarizations are the strongest (i.e. the largest induced dipoles), its orientation (the acceptor-to-donor direction).

Redox potentials. Calculated redox potentials were computed based on a literature method used for Anderson polyoxometalates.[29] Using the ground state optimized geometries for each pair of redox states in acetonitrile solution, subtraction of the Gibbs free energies (sum of electronic and thermal) in solution ($G^{\circ}_{(s)}$), e.g. $G^{\circ}_{(s, -3)}$ - $G^{\circ}_{(s, -2)}$ gives the free energy change $\Delta G^{\circ}_{(s, -2/-3)}$ in solution upon reduction. Using the thermodynamic relationship between the standard free energy change and the standard cell potential (ΔG°= -nFE°_{cell}) rearrangement and substitution of our computed value of $\Delta G^{\circ}_{(s, ox/red)},$ yields the value of $E^{\circ}_{cell.}$. To obtain the redox potential versus our reference - Ferrocene/Ferrocenium (Fc/Fc^+) the standard cell potential of the standard hydrogen electrode (SHE) (4.44V) and the potential of our reference in acetonitrile versus the SHE (0.624 V)[30] were subtracted from the computed value of E°_{cell}. These calculations do not take into account interaction with cations, or explicit solvent molecules, which are required for highly accurate estimation of potentials in highly charged species.[31]

Computer codes. All DFT and TD-DFT calculations were performed using the Gaussian16 package.[32] The molecular structures, molecular orbitals and electron density difference plots were generated using GaussView.[33] Simulated UV/vis absorption spectra were generated using DrawSpectrum software,[34] and USR pictures were generated using DrawMol software.[35]

Table S5. Selected computed bond lengths (Å) and angles (°) of anions $[\mathbf{1}]^{2-/3-}$. Mo^{im} – imido bearing Mo; O - central O atom in the POM. Alkyne twist angle refers to means the angle between the two six-membered rings on each side of the alkyne.

Anion	Mo^{im}-N^{im}	N^{im}-C	O-Mo^{im}-N^{im}	Mo^{im}-N^{im}-C	Alkyne twist angle
$[\mathbf{1}]^{2-}$<br>$[\mathbf{1}]^{3-}$	1.726<br>1.740	1.374<br>1.376	175.8<br>179.2	176.8<br>175.2	0.74<br>4.03

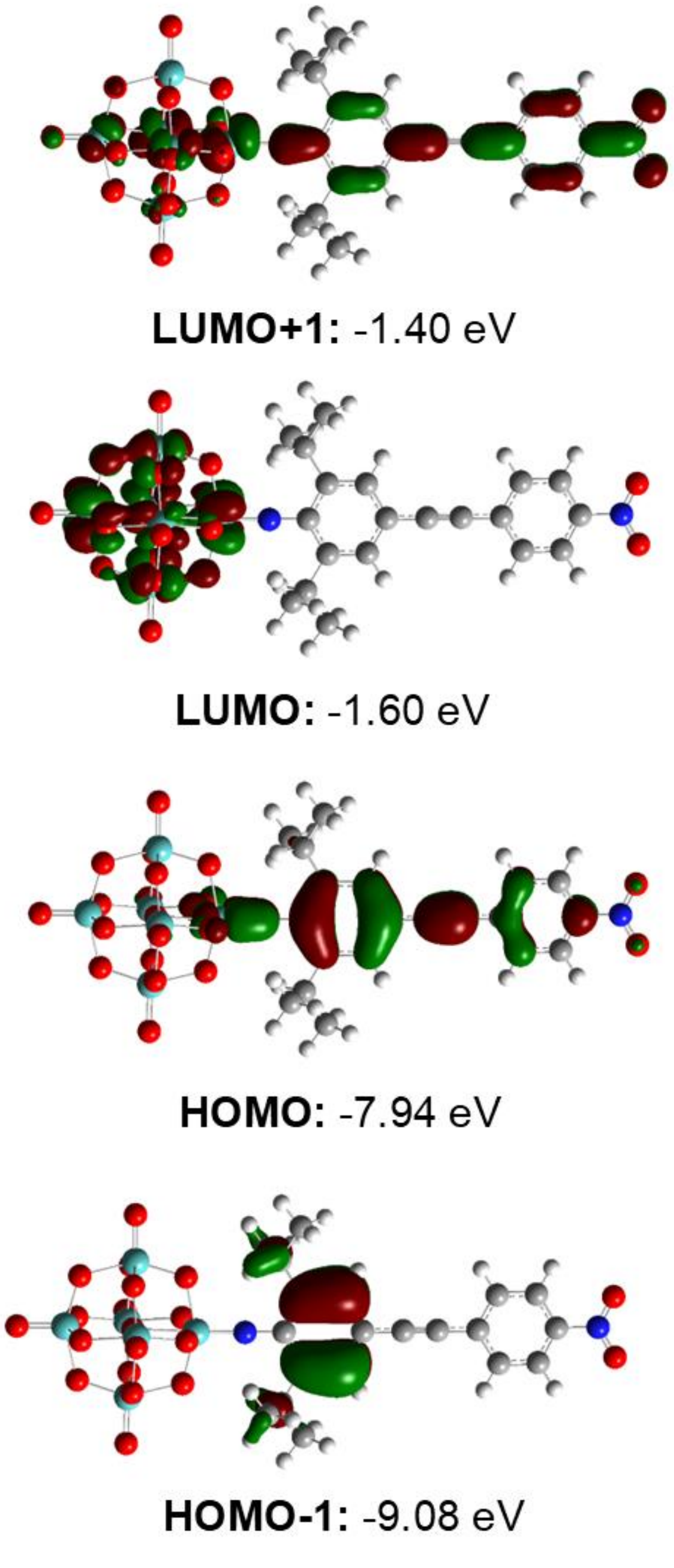

Figure S6 HOMO/HOMO-1 and LUMO/LUMO+1 orbitals of oxidised state $[\mathbf{1}]^{2-}$ calculated at the IEF-PCM(acetonitrile)/TD-DFT/ωB97X-D/6-311G(d)/LanL2TZ level of theory.

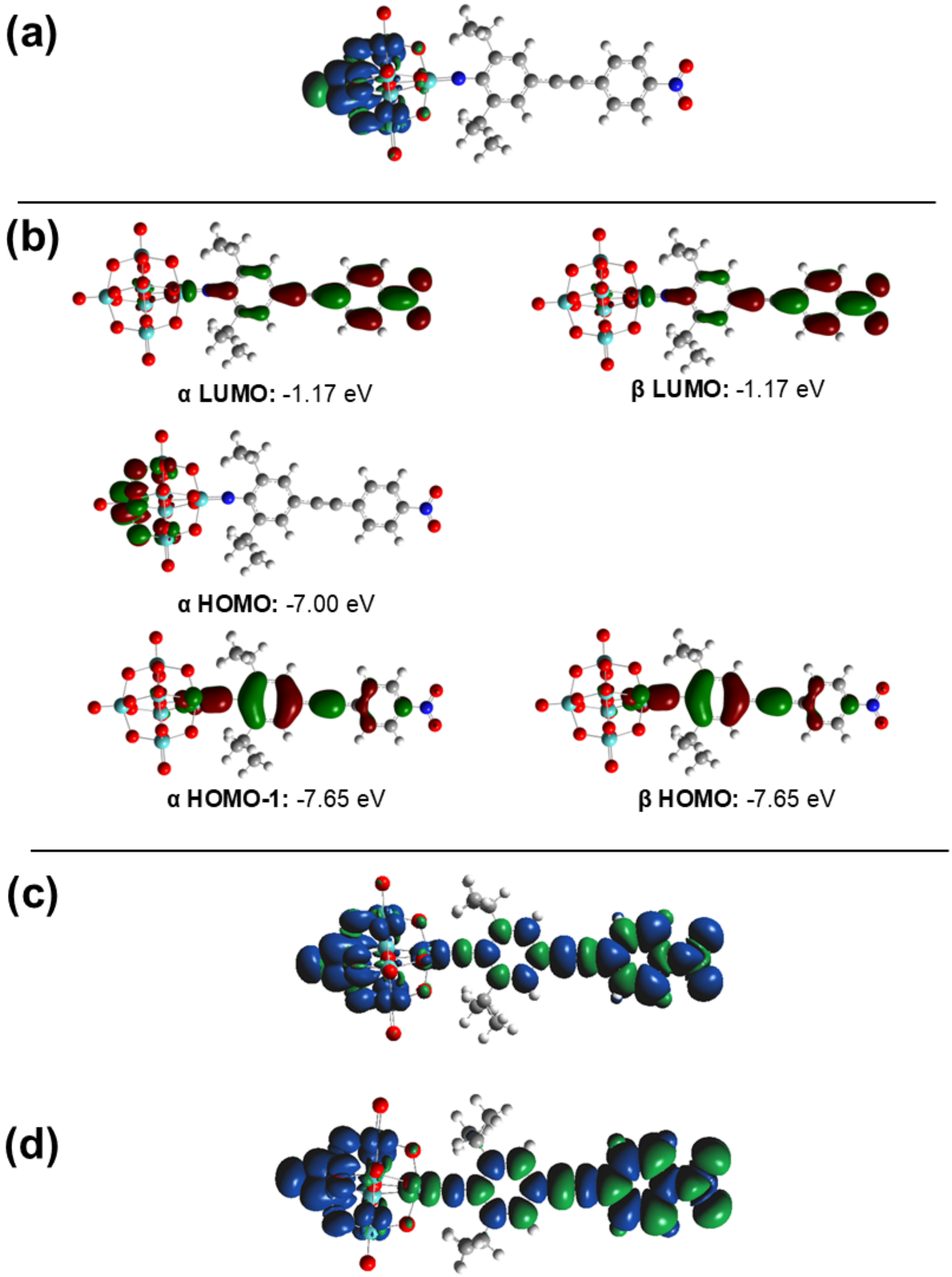

Figure S7 Computed electronic structures of reduced species: **(a)** Spin density of [**1**]$^{3-}$ (isovalue = 0.03 a.u.). **(b)** HOMO/HOMO-1 and LUMO orbitals of reduced species [**1**]$^{3-}$. (**c**) Spin density of [**1**]$^{4-}$, triplet S = 1 (isovalue = 0.03 a.u.). (**d**) Spin density of [**1**]$^{4-}$, singlet S = 0 (isovalue = 0.03 a.u.). All calculated at the IEF-PCM(acetonitrile)TD-DFT/ωB97X-D/6-311G(d)/LanL2TZ level of theory.

Table S6. Energies, spin contaminations $\hat{S}^2$, oscillator strengths f_{GE}, charge transfer distances d_{CT}, variations of dipole moment upon excitation from the GS to the nth dominant lowest-energy excited state ($\Delta\mu_{GE}$), and two-state approximation (TSA) estimations of associated contributions β of the both oxidized $[\mathbf{1}]^{2-}$ and reduced $[\mathbf{1}]^{3-}$ species as calculated at the IEFPCM(acetonitrile) TD-DFT/ωB97X-D/6-311G(d)/LanL2TZ level of approximation. The nonequilibrium solvation approach was adopted.

Anion	n	ΔE_{GE} (eV)	λ_{GE} (nm)	$\hat{S}^2$	f_{GE}	d_{CT} (Å)	$\Delta\mu_{GE}$[a] (D)	$\beta_{zzz}^{TSA} = \frac{9}{2}\frac{f\,\Delta\mu}{\Delta E^3}$ (10^{-30} esu)	$\lvert\Sigma\beta_{zzz}^{TSA}\rvert$ (10^{-30} esu)
$[\mathbf{1}]^{2-}$	2	3.34	371	0	1.84	0.88	+2.31	+35.13	31.81
	10	4.05	306	0	0.18	1.47	-3.98	-3.32	
$[\mathbf{1}]^{3-}$	14	3.20	382	0.76	2.26	0.86	-2.10	-44.58	51.04
	43	4.00	310	0.83	0.19	2.90	-7.06	-6.46	

[a]Defined as $\Delta\mu_{GE} = \mu_{ES} - \mu_{GS}$. With z defined by the molecular axis (POM to -NO_2), a positive $\Delta\mu_{GE}$ thus indicates charge transfer towards the POM as increased negative charge at the POM will increase μ.

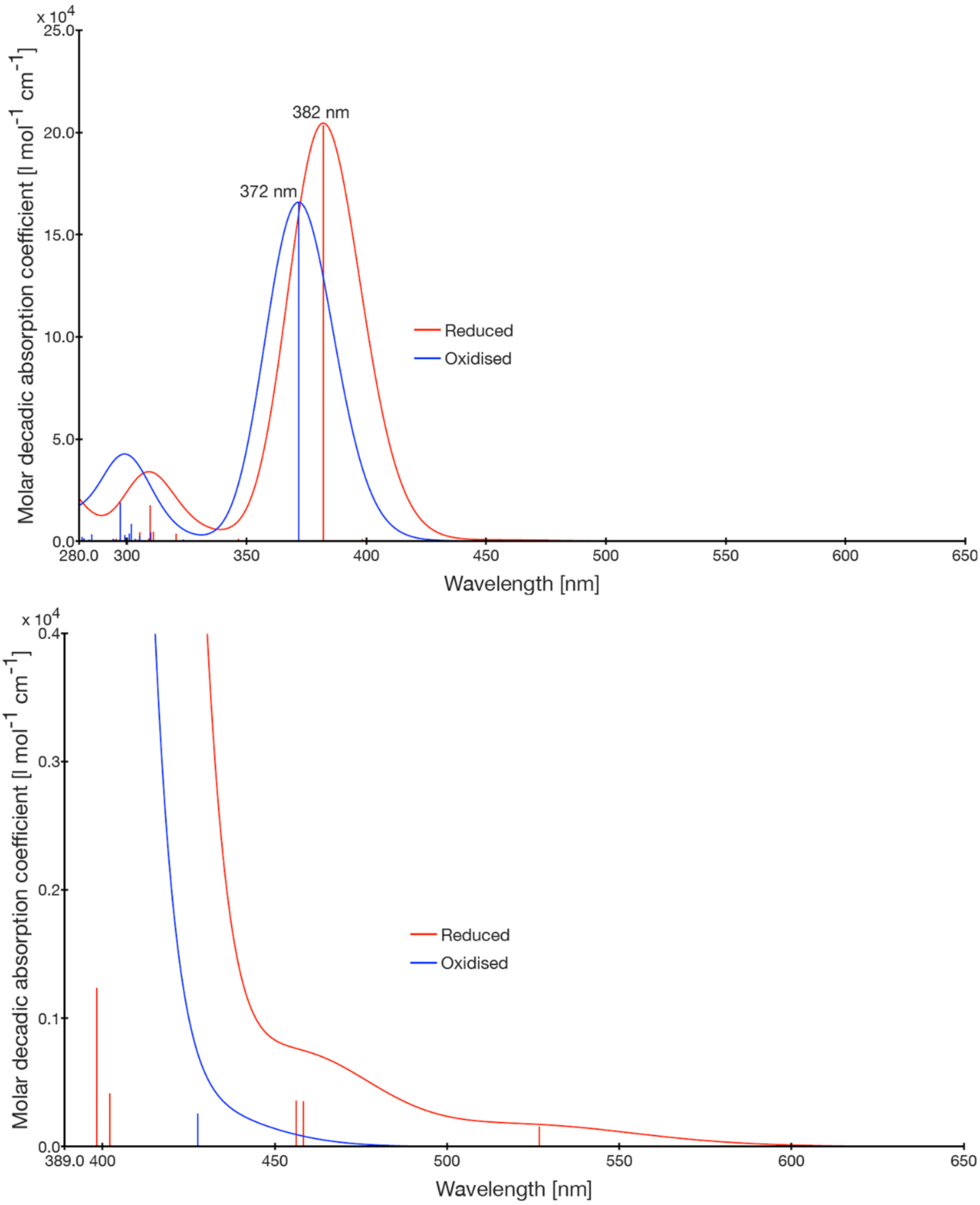

Figure S8 Computed absorption spectra of the oxidized form $[\mathbf{1}]^{2-}$ and reduced form $[\mathbf{1}]^{3-}$ as calculated at the IEFPCM (solvent = acetonitrile) TD-DFT/ωB97X-D/6-311G(d)/LanL2TZ level of approximation. FWHM = 0.3 eV. 300 excited states have been calculated.

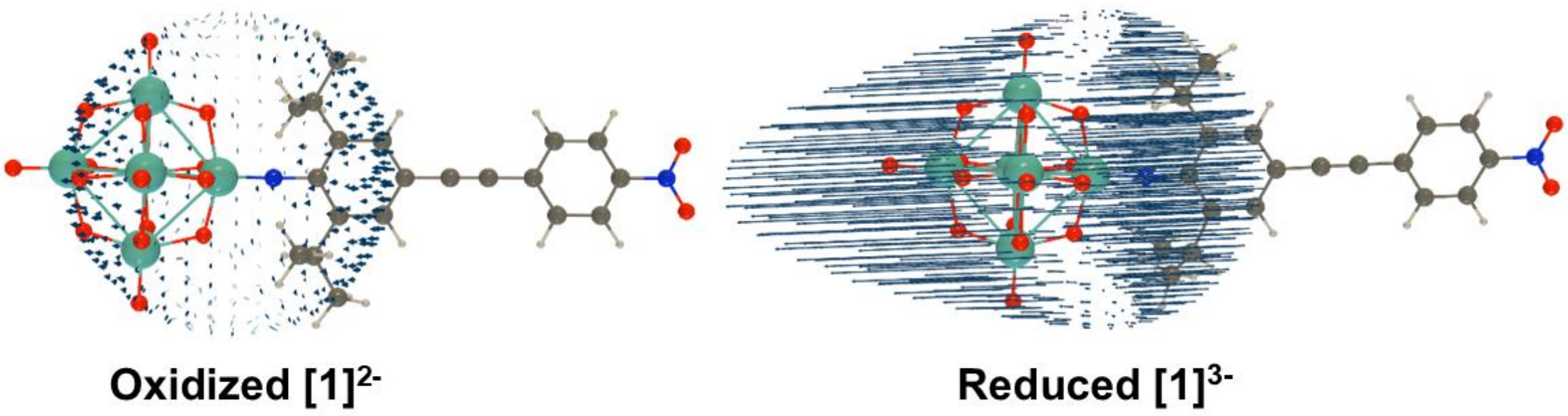

Figure S9 Unit sphere representation (USR) of the first hyperpolarizability tensor (λ = 1064 nm) of oxidized (off) [**1**]$^{2-}$ and reduced (on) [**1**]$^{3-}$ anions as calculated at the IEFPCM(solvent = acetonitrile) TD-DFT/ωB97X-D/6-311G(d)/LanL2TZ level of approximation. Factor = 10^{-4} Å/a.u. β.

EPR Spectroscopy

Methods and Instrumentation. A sample of [**1**]$^{3-}$ was prepared by bulk electrolysis of $[NBu_4]_2$[**1**] (0.07 mM) in 0.1 M $[NBu_4][BF_4]$ electrolyte in acetonitrile. This was syringed into standard 4 mm quartz EPR tubes (Wilmad) pre-purged with argon and then rapidly frozen in liquid nitrogen. EPR spectra were obtained on a Bruker E560 EleXsys spectrometer equipped with a standard Bruker EPR cavity (ER4123 SHQE) and an Oxford instruments flow cryostat (ESR900) using liquid helium (10 K). Experimental parameters: microwave power, 3.15 µW; field modulation frequency, 100 kHz; field modulation amplitude, 0.8 mT. All spectral simulations were performed using the MATLAB-based EasySpin package[36] Data presented for $[Mo_6O_{19}]^{3-}$ were published previously.[37]

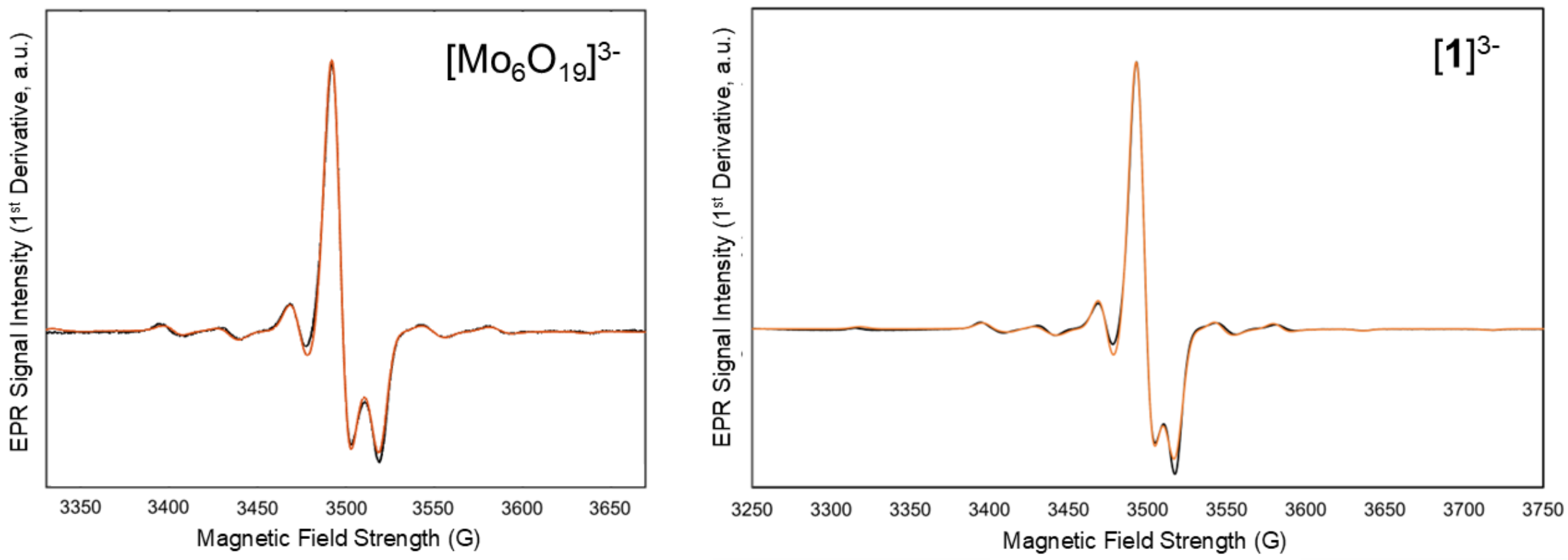

Figure S10 X-band EPR spectra of $[Mo_6O_{19}]^{3-}$ and arylimido derivative [**1**]$^{3-}$ at 10 K. Data – black trace; Fit – orange trace.

Table S7 Parameters extracted from fit of 10 K X-band EPR data for $[Mo_6O_{19}]^{3-}$ and $[\mathbf{1}]^{3-}$.

	g_x	g_y	g_z	A_x / MHz	A_y / MHz	A_z / MHz	Isotropic linewidth / G
$[Mo_6O_{19}]^{3-}$	1.9123 ± 0.0055	1.9258 ± 0.0059	1.9258 ± 0.0073	209.30	99.91	93.46	1.00
$[\mathbf{1}]^{3-}$	1.9170 ± 0.0056	1.9270 ± 0.0050	1.9300 ± 0.0084	212.30	89.21	98.88	0.95

The spectra (Figure S8) show hardly any difference between $[Mo_6O_{19}]^{3-}$ and $[\mathbf{1}]^{3-}$. Previously with a more electron donating ($^iPr_2PhNMe_2$) imido-aryl ligand,[37] a small shift towards lower field *vs* $[Mo_6O_{19}]^{3-}$ was seen in the *ca*. 3520 G peak – this is barely seen in $[\mathbf{1}]^{3-}$, consistent with the electron withdrawing -$CCPhNO_2$ group in the 4-position in place of -NMe_2. The line width is virtually identical to that of $[Mo_6O_{19}]^{3-}$, and there is no evidence of an interaction between the electron and nitrogen nuclear spin, consistent with the DFT calculated SOMO based predominantly on the other side of the $\{Mo_6\}$ unit to the imido group (Figure S8a).

Spectroelectrochemistry

All measurements were performed using a Spectroelectrochemistry Reading RT OTTLE cell,[38] with either an Agilent Cary 60 UV-vis spectrophotometer and Autolab μ-III potentiostat, or an Agilent Cary 5000 UV-vis spectrophotometer and an Autolab PGStat 302 potentiostat/galvanostat. The analyte concentration was *ca.* 0.8×10^{-3} M in 0.3 M NBu_4BF_4 in dry acetonitrile for measurements performed on the Cary 60, and *ca.* 5×10^{-3} M for the longer-range measurements performed on the Cary 5000. A reductive potential of between -0.8 and -0.5 V vs Ag/Ag^+ was applied for between two and five minutes, varied to account for potential drift determined by a cyclic voltammogram taken immediately prior. UV-vis spectra were recorded at 15 second intervals. When no further changes were observed, a re-oxidising potential of -0.3 to 0 V vs Ag/Ag^+ was applied for between two and five minutes until the continuous UV-vis monitoring showed no further changes.

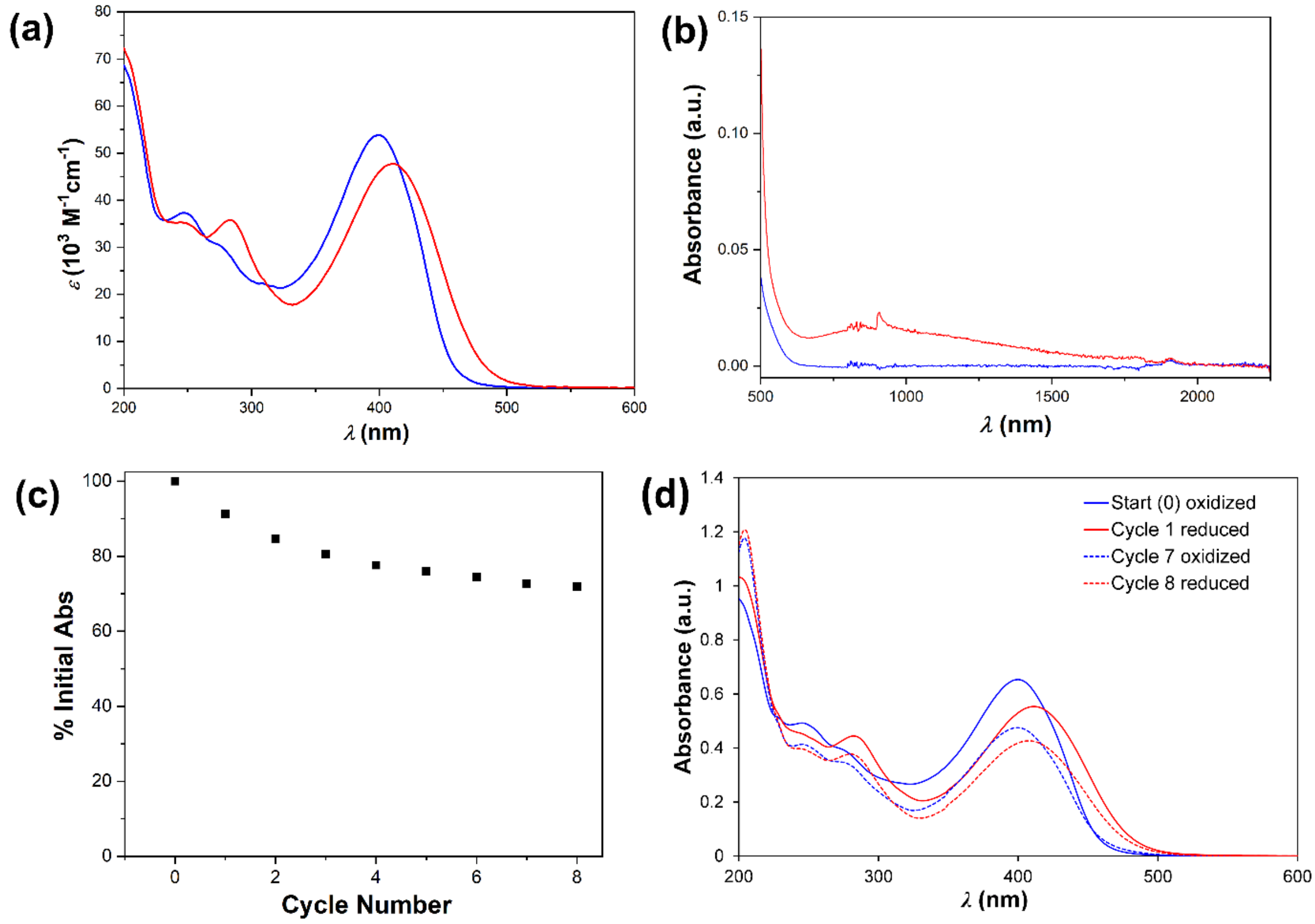

Figure S11 **(a)** UV-vis (left) spectrum of $[NBu_4]_2$[**1**] before (blue) and after (red) electrochemical reduction (concentration *ca.* 0.8 × 10^{-3} M in 0.3 M NBu_4BF_4). **(b)** UV-vis-NIR spectrum of $[NBu_4]_2$[**1**] before (blue) and after (red) electrochemical reduction**.** **(c)** Decline in peak absorbance of the LPCT peak of $[NBu_4]_2$[**1**] (left) over eight cycles of electrochemical reduction and reoxidation (concentration *ca.* 0.8 × 10^{-3} M in 0.3 M NBu_4BF_4). (**d**) Selected underlying spectra supporting (**c**), showing spectra before and after the first and final reduction cycles.

Hyper-Rayleigh Scattering (HRS)

Measurement of HRS. General details of the hyper-Rayleigh scattering (HRS) experiment have been discussed elsewhere,[39] and the experimental procedure and data analysis protocol used for the fs measurements used in this study were as previously described.[40] Measurements were carried out in acetonitrile dried over activated 3Å molecular sieves, and degassed with N_2. The solutions were dilute (*ca.* 10^{-5} M) and filtered (Millipore, 0.45 μm), such that self-absorption of the SHG signal and light scattering was negligible, verified by the linear relation between signal and a series of five concentrations. UV-vis spectra before and after HRS indicated no noticeable decomposition resulting from two photon absorption. The 1064 nm source was a Spectra-Physics InSight® DS+ laser (1W average power, sub-100 fs pulses, 80 MHz). In this setup, the collection optics are coupled to a spectrograph (model Bruker 500is/sm), together with an EMCCD camera (Andor Solis model iXon Ultra 897). Correction for multiphoton induced fluorescence was done by subtracting the broad MPF background signal from the narrow HRS peak (FWHM ± 9 nm). The high accuracy of this setup enables us to use the solvent as an internal reference (acetonitrile, $\beta_{HRS,1064}$ = 0.258 × 10^{-30} esu; $\beta_{zzz,1064}$

= 0.623 × 10^{-30} esu).[41] For consistency, the B convention is used to report all β values, both experimental and computational.

It is noteworthy that measurements previously conducted on the isopropyl free analogue [**1H**]$^{2-}$ found no measureable HRS activity.[7] This may be a consequence of measurement conditions as the -NO_2 derivatives show two-photon fluorescence which can mask HRS signal. Shifts in wavelength of the fluorescence, and changes of intensity of both signals as a result of the structural change, coupled with a new optical set up better able to discriminate fluorescence, are likely responsible for these different results.

In-situ Electrochemistry-HRS. Single-cycle measurements were performed using a custom-designed two compartment cell with the working compartment constructed from a 1 cm path length optical quality Pyrex fluorescence cuvette as described previously.[37] Multi-cycle redox switching measurements were performed using a modified three-compartment cell (Figure S11). In both cells Pt gauze was used as the working electrode, Ag wire as the reference, and a Pt wire coil as the counter. A separate Pt wire electrode was added to the Pt gauze working electrode set, which could be connected in place of the gauze to enable more accurate *in situ* cyclic voltammetry and determine potentials for bulk electrolysis. Analyte concentrations were *ca* 10^{-4} M in 0.2 or 0.3 M [NBu_4][BF_4]: this high concentration was needed for electrochemical performance and to ensure an adequate difference between the sample and solvent/electrolyte baseline. HRS signal was recorded every 5 seconds as described above, with 10 measurements taken before applying potential to establish a baseline value for the oxidized states. To achieve switching a reductive electrochemical potential of -1.1 V (single off-on-off cycle) or -0.8 V (multiple cycle off-on-off cycles) vs Ag/Ag^+ was applied using Princeton Applied Research Parstat 2273 or Autolab PGStat 302 potentiostat for 400 seconds before re-oxidation with a potential of 0 V vs Ag/Ag^+.

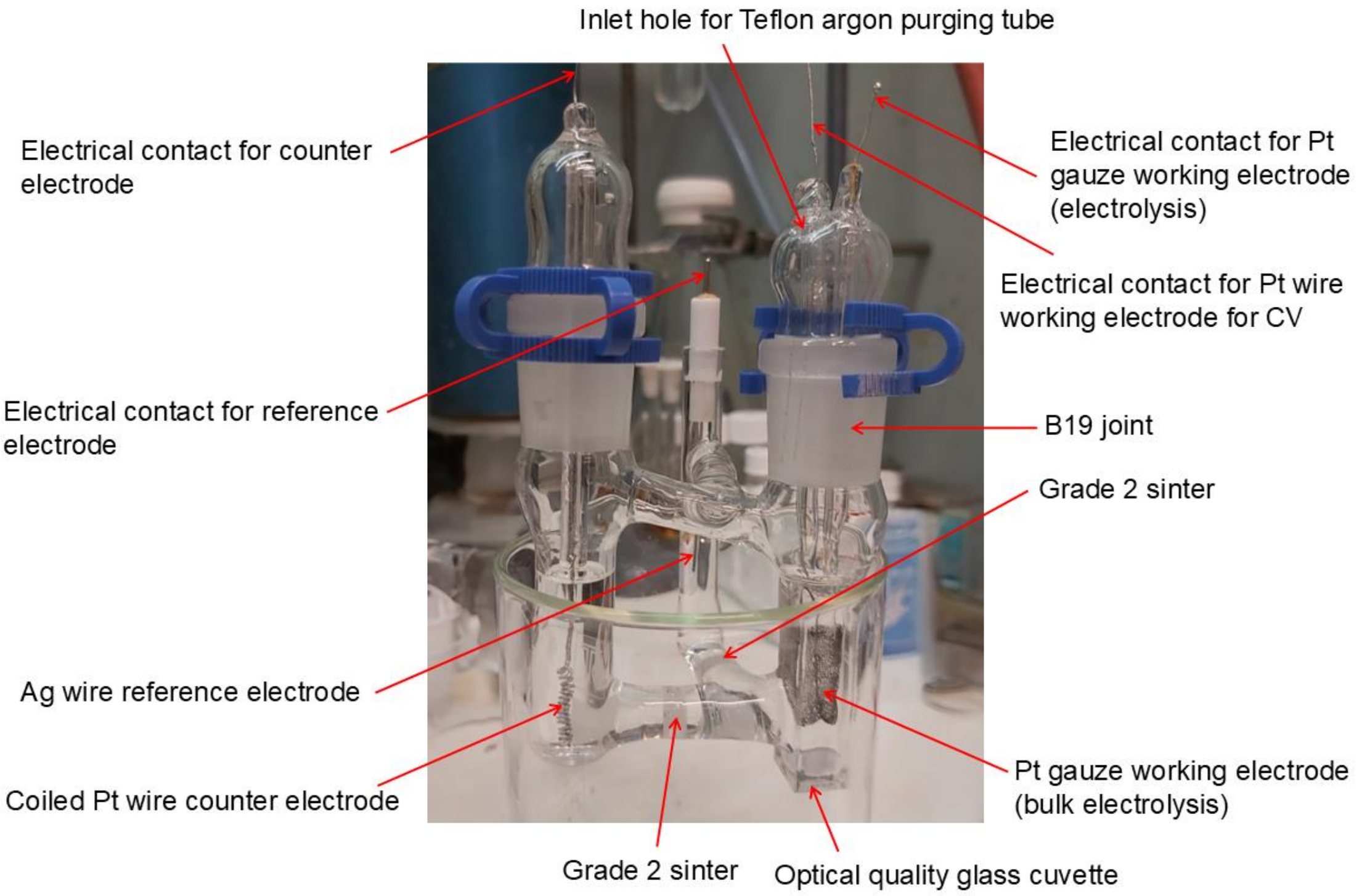

Figure S12 Modified Electrochemistry-HRS three compartment cell with an additional Pt wire electrode for in situ cyclic voltammetry (disconnected during bulk electrolysis).

Possible decomposition / over-reduction products and their effect on HRS signal. The bulk electrolysis (BE) and spectroelectrochemistry measurements, and imperfect HRS switching make it clear that reduced anion [**1**]$^{3-}$ is not completely stable. Nonetheless, post-BE analysis (Fig. S4) indicates that at least 75% of [**1**]$^{2-/3-}$ persists at the end of the first reduction cycle, and the β-values of likely decomposition (**1L**) or over-reduction products ([**1R**]$^{2-/3-}$, Fig. S13) will be too low to explain the observed signal if present at 25% (or even 100%) of the initial concentration of $[NBu_4]_2$[**1**], especially considering HRS signal ∝ β^2. This is based on comparison with known analogues measured using the same technique (Fig. S13), and well-established structure activity relationships seen in both organic and POM chromophores.

Moreover, rather than increasing upon reduction, signal associated with D-π-A products such as **1L** and [**1R**]$^{2-/3-}$ will either be unchanged or decrease at the more negative potential.[4,37,42] This is confirmed by computation – for [**1R**]$^{2-}$ we obtain $\beta_{1064,zzz}$= 301 × 10^{-30} esu for [**1R**]$^{2-}$, and only 16 × 10^{-30} esu for [**1R**]$^{3-}$. Thus, while presence of decomposition products may contribute to the increased off state signals observed over multiple cycles, the switch-on is due only to [**1**]$^{3-}$, and β-values estimated for [**1**]$^{3-}$ must be conservative – they are made based on experimental signal assuming 100% conversion to [**1**]$^{3-}$, but 100% conversion to [**1**]$^{3-}$ does not occur and other products present are less active. It should be noted that any possible hydroxylamine (N-OH) products would have lower β than for [**1R**]$^{2-}$ due to their weaker capacity vs anilines as donors, while arylimido-free molybdate decomposition products (e.g. $Mo_6O_{19}^{2-/3-}$, $Mo_2O_7^{2-}$, $Mo_8O_{26}^{2-}$) are NLO inactive: they are symmetric, and O→Mo CT transitions are high energy with small dipole moment changes. Over-reduction to [**1**]$^{4-}$ is

unlikely on the basis of potentials applied and charges passed (see below), such a species would likely be less stable than [**1**]$^{3-}$, and no evidence of nitro-radical species was seen in EPR (Figure S10) where [**1**]$^{3-}$ was electrochemically generated at similar potential to our cycled electrochemistry-HRS. There is no existing literature on 2nd order NLO properties for such D-π-D diradical systems.

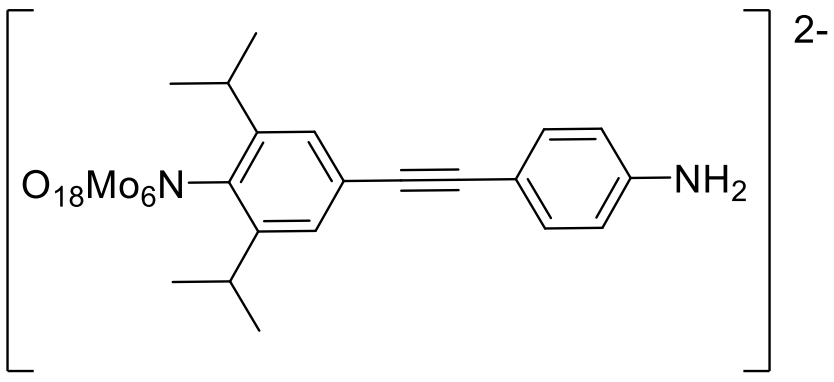

Nitro reduction product [1R]$^{2-}$

$[Mo_6O_{18}N(iPr)_2PhCCPhNH_2]^{2-}$

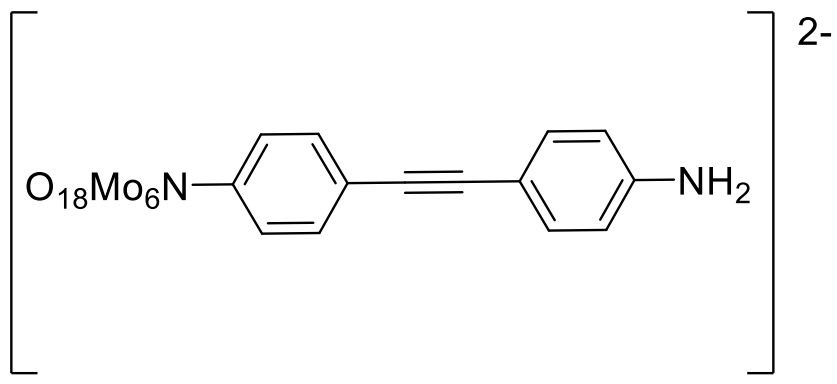

Closest measured analogue [1HR]$^{2-}$

$[Mo_6O_{18}NPhCCPhNH_2]^{2-}$

$\beta_{1064,zzz}$ **= 372 x 10^{-30} esu (HRS)**

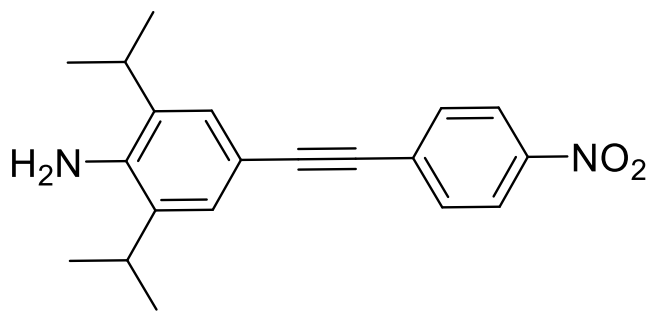

Decomposition product 1L

4-[2-(4-nitrophenyl)ethynyl]-2,6-diisopropylbenzenamine

$H_2N(^iPr)_2PhCCPhNO_2$

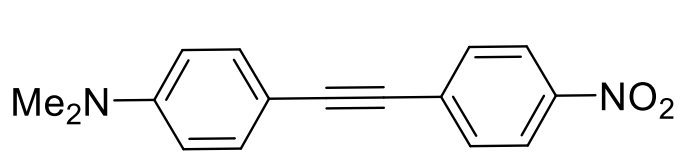

Closest measured analogue 1HMeL

4-[2-(4-nitrophenyl)ethynyl]-benzenamine

$H_2NPhCCPhNO_2$

$\beta_{1064,zzz}$ **= 452 x 10^{-30} esu (HRS)**

Figure S13 Possible NLO active nitro-reduction product [**1R**]$^{2-}$, decomposition product **1L** and their closest HRS measured analogues. Known structure activity relationships indicate that the β-values for [**1R**]$^{2-}$ and **1L** will be similar or lower than for the analogues, and thus substantially lower than that extracted for [**1**]$^{3-}$ ($\beta_{1064,zzz} = 670 \times 10^{-30}$ esu).

Additional experimental considerations and observations support [**1**]$^{3-}$ being the source of on-state signal, and argue against generation of imido-POM byproducts:

- The potential used in the cycled experiment (main paper Fig. 4a) was carefully chosen to make sure only the first reduction of $[NBu_4]_2$[**1**] could be accessed. UV-vis spectra of the reduced state generated under these conditions match those of [**1**]$^{3-}$ obtained in the OTTLE spectroelectrochemistry cell (Figure S11).
- The cycle 1 "on" signal enhancements with sufficient charge passed to generate 60% [**1**]$^{3-}$ (9.7× expected, *ca.*10× observed, main paper Fig. 4a) and 97% [**1**]$^{3-}$ (15.1× expected, *ca.* 15× observed, main paper Fig. 4b) scale almost exactly as expected. This direct correlation between charge passed, and signal is consistent with the 1e$^-$ reduction product being responsible for signal at both potentials.
- Signal increases instantaneously upon application of the reducing potential, and the rate of increase attenuates with time, consistent with known constant potential electrolysis kinetics. If the main active species were generated by decomposition after the initial electron transfer, a delay or induction period would occur before signal increase.

- Even at the more negative potential, charges passed were too small to generate large amounts of multi-electron reduction products – the total charge passed in Fig. 4b could convert at most 15% of $[\mathbf{1}]^{2-}$ to an aniline ($6e^-$, $6H^+$).
- Post-bulk electrolysis CV (Figure S4) shows a decline in the current for the imido-POM based $[\mathbf{1}]^{2-/3-}$ reduction and only emergence of a very small peak for $[Mo_6O_{19}]^{2-/3-}$ (*ca.* -0.8V vs $Fc^{0/+}$). As POM redox potentials for $[Mo_6O_{18}NArCCAr]^{2-}$ derivatives vary little with substitution of the aryl group more distant from the POM,[7,42] this is consistent with loss of imido-Mo_6 moieties and generation of molybdate species such as $[Mo_2O_7]^{2-}$, previously shown for the reduction of another $[Mo_6(^iPr)_2PhCCAr]^{2-}$ system,[42] rather than generation of any new imido-POM derivatives. Small molybdates such as $[Mo_2O_7]^{2-}$ are electrochemically inactive in the window studied. Changes to the nitro-based reduction peak are smaller, and the reduction potential of 4-nitro-diphenylacetylene has been reported within 20 mV of that we observe for the $[\mathbf{1}]^{3-/4-}$ nitro reduction.[43] Thus, an $-NO_2$ substituted diphenylacetylene – likely **1L** – must persist.
- Multi-cycle spectroelectrochemistry (Fig. S11c,d) shows a smaller UV-visible response to reduction in the latter cycles, consistent with generation of decomposition products that are not electroactive at the applied potential (i.e. $[Mo_2O_7]^{2-}$, nitro-diphenylacetylenes). The similar position of the lower energy (*ca.* 400 nm) band of the oxidized spectrum after 7 cycles to the starting spectrum, but lowered absorbance, is consistent with production of **1L** as related structures have CT bands at around this wavelength, but significantly lower extinction coefficients than imido-POM derivatives.[7,44]

Considering all of the evidence, including previously observed decomposition pathways for reduced POM imido species, the likeliest decomposition product is thus **1L**, and NLO inactive small molybdates. **1L** could contribute to the rising background "off" signal observed, but cannot be responsible for the redox switching effect.

References

1. W. L. F. Armarego, C. L. L. Chai, *Purification of laboratory chemicals*; 6th ed.; Elsevier/Butterworth-Heinemann: Amsterdam; Boston, 2009.

2. W. G. Klemperer, *Inorg. Synth.* **1990**, *27*, 71.

3. B. R. D'Souza, T. K. Lane, J. Louie, *Org. Lett*., **2011**, *13*, 2936.

4. B. R. Hood, Y. de Coene, C. F. Jones, N. Deveaux, J. M. Barber, C. G. Marshall, C. A. Jordan, N. R. Halcovitch, B. Champagne, K. Clays and J. Fielden, *Inorg. Chem.* **2025**, *64*, 8408.

5. L. Xu, M. Lu, B. Xu, Y. Wei, Z. Peng, D. R. Powell, *Angew. Chem. Int. Ed.* **2002**, *41*, 4129.

6. A. Al-Yasari, N. Van Steerteghem, H. El Moll, K. Clays, J. Fielden, *Dalton Trans.*, **2016**, *7*, 2818.

7. A. Al-Yasari, N. Van Steerteghem, H. Kearns, H. El Moll, K. Faulds, J. A. Wright B. S. Brunschwig, K. *Clays, Inorg. Chem.*, **2017**, *56*, 10181.

8. A. Al-Yasari, P. Spence, H. El Moll, N. Van Steerteghem, P. N. Horton, B. S. Brunschwig, K. Clays, J. Fielden, *Dalton Trans.*, **2018**, *47*, 10415-10419.

9. *CrysAlisPro* (Version 1.171.40.68a), Rigaku Oxford Diffraction, Rigaku Corporation, Tokyo, Japan, **2019**.

10. G. M. Sheldrick, *Acta Cryst. A*, **2015**, *71*, 3.

11. O. V. Dolomanov, L. J. Bourhis, R. J. Gildea, J. A. K. Howard, H. Puschmann, *J. Appl. Crysallogr.* **2009**, *42*, 339.

12. G. M. Sheldrick, *Acta. Cryst. C*, **2015**, *71*, 3.

13. S. K. Ibrahim, Ph.D Thesis, University of Sussex, **1992**.

14. J.D. Chai and M. Head-Gordon, *Phys. Chem. Chem. Phys.* **2008**, *10*, 6615.

15. A.D. Becke, *J. Chem. Phys*. **1997**, *107*, 8554.

16. R. Krishnan, J.S. Binkley, R. Seeger, J.A. Pople, *J. Chem. Phys.* **1980**, *72*, 650.

17. L.E. Roy, P.J. Hay, R.L. Martin, *J. Chem. Theory Comput.* **2008**, *4*, 1029.

18. E. Rtibi, M. Abderrabba, S. Ayadi, B. Champagne, *Inorg. Chem*. **2019**, *58*, 11210.

19. J. Tomasi, B. Mennucci, R. Cammi, *Chem. Rev*. **2005**, *105*, 2999.

20. M.E. Casida, in "Recent advances in Density Functional Theory", edited by D.P. Chong, Singapore, World Scientific (1995), pp 155-192.

21. T. Le Bahers, C. Adamo, I. Ciofini, *J. Chem. Theory Comput.* **2011**, *7*, 2498.

22. S.J.A. Van Gisbergen, J.G. Snijders, E.J. Baerends, *J. Chem. Phys*. **1998**, *109*, 10644.

23. T. Helgaker, S. Coriani, P. Jørgensen, K. Kristensen, J. Olsen, K. Ruud, *Chem. Rev.* **2012**, *112*, 543.

24. B. Champagne, P. Beaujean, M. de Wergifosse, M. Cardenuto, V. Liégeois, F. Castet, in Frontiers of Quantum Chemistry, edited by M. Wojcik, H. Nakatsuji, B. Kirtman, Y. Ozaki, Springer, Singapore, 2018, pp 117-138.

25. M. de Wergifosse, B. Champagne, *J. Chem. Phys.* **2011**, *134*, 074113.

26. L.E. Johnson, L.R. Dalton, B.H. Robinson, *Acc. Chem. Res*. **2014**, *47*, 3258.

27. K. Garrett, X.A. Sosa Vazquez, S.B. Egri, J. Wilmer, L.E. Johnson, B.H. Robinson, C.M. Isborn, *J. Chem. Theory Comput.* **2014**, *10*, 3821.

28. A. Tuer, S. Krouglov, R. Cisek, D. Tokarz, V. Barzda, *J. Comput. Chem.* **2011**, *32*, 1128.

29. S. Schönweiz, M. Heiland, M. Anjass, T. Jacob, S. Rau, C. Streb, *Chem. Eur. J.* **2017**, *23*, 15370.

30. V. V. Pablishchuk, A.W. Addison*, Inorg. Chim. Acta* **2000**, *298*, 97.

31. J. A. Thompson, R. González-Cabaleiro, L. Vilà-Nadal, *Inorg. Chem.* **2023**, *62*, 12260.

32. M.J. Frisch, G.W. Trucks, H.B. Schlegel, G.E. Scuseria, M.A. Robb, J.R. Cheeseman, G. Scalmani, V. Barone, G.A. Petersson, H. Nakatsuji, X. Li, M. Caricato, A. V Marenich, J. Bloino, B.G. Janesko, R. Gomperts, B. Mennucci, H.P. Hratchian, J. V Ortiz, A.F. Izmaylov, J.L. Sonnenberg, D.

Williams-Young, F. Ding, F. Lipparini, F. Egidi, J. Goings, B. Peng, A. Petrone, T. Henderson, D. Ranasinghe, V.G. Zakrzewski, J. Gao, N. Rega, G. Zheng, W. Liang, M. Hada, M. Ehara, K. Toyota, R. Fukuda, J. Hasegawa, M. Ishida, T. Nakajima, Y. Honda, O. Kitao, H. Nakai, T. Vreven, K. Throssell, J.A. Montgomery Jr., J.E. Peralta, F. Ogliaro, M.J. Bearpark, J.J. Heyd, E.N. Brothers, K.N. Kudin, V.N. Staroverov, T.A. Keith, R. Kobayashi, J. Normand, K. Raghavachari, A.P. Rendell, J.C. Burant, S.S. Iyengar, J. Tomasi, M. Cossi, J.M. Millam, M. Klene, C. Adamo, R. Cammi, J.W. Ochterski, R.L. Martin, K. Morokuma, O. Farkas, J.B. Foresman, D.J. Fox, Gaussian16 Revision A.03, **2016**.

33. R. Dennington, T. A. Keith, J. M. Millam, GaussView Version 6.1, Semichem Inc, Shawnee Mission KS, **2016**.

34. DrawSpectrum, V. Liégeois, U. Namur, www.unamur.be/drawspectrum.

35. DrawMol, V. Liégeois, U. Namur, www.unamur.be/drawmol.

36. S. Stoll, A. Schweiger, EasySpin, a comprehensive software package for spectral simulation and analysis in EPR. *J. Magn. Reson.* **2006**, *178*, 42-55.

37. B. R. Hood, Y. de Coene A. V. Torre Do Vale Froes, C. F. Jones, P. Beaujean, V. Liégois, F. MacMillan, B. Champagne, K. Clays, J. Fielden, *Angew. Chem. Int. Ed.* **2023**, *62*, e202215537.

38. M.Krejčik, M.Daněk, F.Hartl, *J. Electroanal. Chem.* **1991**, *317*, 179.

39. (a) K. Clays, A. Persoons, *Phys. Rev. Lett*. **1991**, *66*, 2980. (b) K. Clays, A. Persoons, *Rev. Sci. Instrum*. **1992**, *63*, 3285. (c) E. Hendrickx, K. Clays, A. Persoons, *Acc. Chem. Res.* **1998**, *31*, 675.

40. (a) G. Olbrechts, R. Strobbe, K. Clays, A. Persoons, *Rev. Sci. Instrum*. **1998**, *69*, 2233. (b) G. Olbrechts, K. Wostyn, K. Clays, A. Persoons, *Optics Lett*. **1999**, *24*, 403. (c) K. Clays, K. Wostyn, G. Olbrechts, A. Persoons, A. Watanabe, K. Nogi, X.-M. Duan, S. Okada, H. Oikawa, H. Nakanishi, H. Vogel, D. Beljonne, J.-L. Brédas, *J. Opt. Soc. Am. B* **2000**, *17*, 256. (d) E. Franz, E. C. Harper, B. J. Coe, P. Zahradnik, K. Clays, I. Asselberghs, *Proc. SPIE–Int. Soc. Opt. Eng*. **2008**, *6999*, 699923-1–699923-11.

41. J. Campo, F. Desmet, W. Wenseleers, E. Goovaerts, *Opt. Express* **2009**, *17*, 4587.

42. B. R. Hood, Y. de Coene, C. F. Jones, N. Deveaux, J. M. Barber, C. G. Marshall, C. A. Jordan, N. R. Halcovitch, B. Champagne, K. Clays and J. Fielden, *Inorg. Chem.* **2025**, *64*, 8408.

43. R. E. Sioda, D. O. Cowan, W. S. Koski, *J. Am. Chem. Soc.* **1967**, *89*, 230.

44. L-T. Cheng, W. Tam, S. R. Marder, A. E. Stiegman, G. Rikken, C. W. Spangler, *J. Phys. Chem.* **1991**, *95,* 10643.
